# Transplantation of insulin-producing cells derived from human mesenchymal stromal/stem cells into diabetic humanized mice

**DOI:** 10.1186/s13287-022-03048-y

**Published:** 2022-07-26

**Authors:** Mohamed A. Ghoneim, Mahmoud M. Gabr, Ayman F. Refaie, Sawsan M. El-Halawani, Mohga M. Al-issawi, Batoul L. Elbassiouny, Mai A. Abd El Kader, Amani M. Ismail, Mona F. Zidan, Mary S. Karras, Raghda W. Magar, Sherry M. Khater, Sylvia A. Ashamallah, Mahmoud M. Zakaria, Malgorzata Kloc

**Affiliations:** 1 Urology Department, Urology and Nephrology Center, Mansoura, Egypt; 2 Biotechnology Department, Urology and Nephrology Center, Mansoura, Egypt; 3 Nephrology Department, Urology and Nephrology Center, Mansoura, Egypt; 4 Immunology Department, Urology and Nephrology Center, Mansoura, Egypt; 5 Microbiology and Immunology Research Program, Children’s Hospital 57357, Cairo, Egypt; 6 Pathology Department, Urology and Nephrology Center, Mansoura, Egypt; 7 The Houston Methodist Research Institute, Houston, TX USA; 8 The Houston Methodist Hospital, Houston, TX USA; 9 The University of Texas, M.D. Anderson Cancer Center, Houston TX, USA

**Keywords:** Mesenchymal stromal cells, Differentiation, Transplantation, Humanized mice, Insulin producing cells, Diabetes, Streptozotocin

## Abstract

**Background:**

The purpose of this study was to investigate allogenic immune responses following the transplantation of insulin-producing cells (IPCs) differentiated from human adipose tissue-derived stem cells (hAT-MSCs) into humanized mice.

**Methods:**

hAT-MSCs were isolated from liposuction aspirates obtained from HLA-A2-negative healthy donors. These cells were expanded and differentiated into IPCs. HLA-A2-positive humanized mice (NOG-EXL) were divided into 4 groups: diabetic mice transplanted with IPCs, diabetic but nontransplanted mice, nondiabetic mice transplanted with IPCs and normal untreated mice. Three million differentiated cells were transplanted under the renal capsule. Animals were followed-up to determine their weight, glucose levels (2-h postprandial), and human and mouse insulin levels. The mice were euthanized 6–8 weeks posttransplant. The kidneys were explanted for immunohistochemical studies. Blood, spleen and bone marrow samples were obtained to determine the proportion of immune cell subsets (CD4^+^, CD8^+^, CD16^+^, CD19^+^ and CD69^+^), and the expression levels of HLA-ABC and HLA-DR.

**Results:**

Following STZ induction, blood glucose levels increased sharply and were then normalized within 2 weeks after cell transplantation. In these animals, human insulin levels were measurable while mouse insulin levels were negligible throughout the observation period. Immunostaining of cell-bearing kidneys revealed sparse CD45^+^ cells. Immunolabeling and flow cytometry of blood, bone marrow and splenic samples obtained from the 3 groups of animals did not reveal a significant difference in the proportions of immune cell subsets or in the expression levels of HLA-ABC and HLA-DR.

**Conclusion:**

Transplantation of IPCs derived from allogenic hAT-MSCs into humanized mice was followed by a muted allogenic immune response that did not interfere with the functionality of the engrafted cells. Our findings suggest that such allogenic cells could offer an opportunity for cell therapy for insulin-dependent diabetes without immunosuppression, encapsulation or gene manipulations.

**Graphical Abstract:**

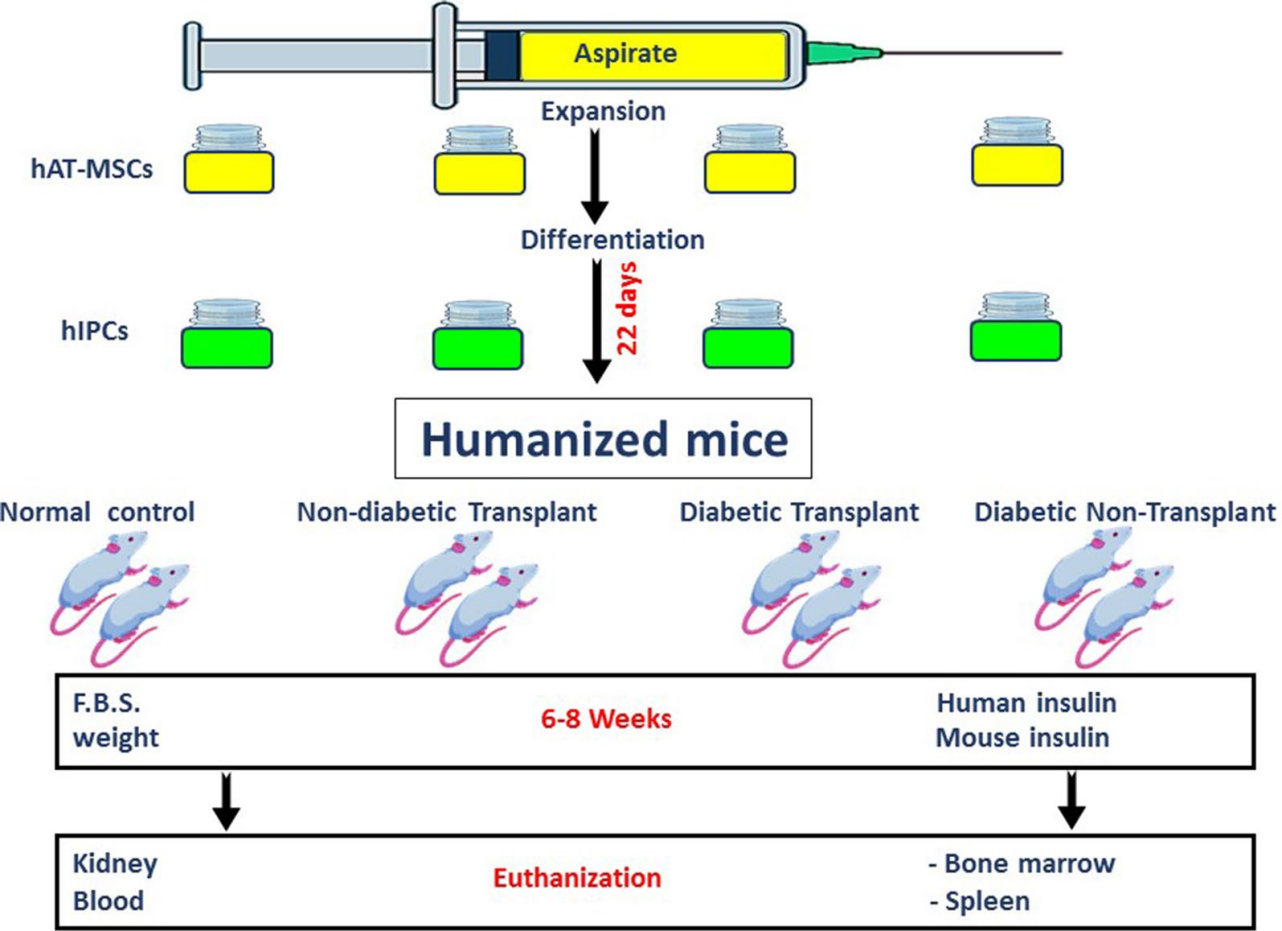

**Supplementary Information:**

The online version contains supplementary material available at 10.1186/s13287-022-03048-y.

## Introduction

Recent progress in the field of regenerative medicine has provided a promising approach for the treatment of diabetes mellitus (DM) through the generation of surrogate β-cells from a variety of stem cell sources. These include embryonic, neonatal, induced pluripotent and mesenchymal/stromal stem cells (MSCs).

The use of MSCs has several advantages. These cells are available from many tissues and can be readily expanded in vitro with a doubling time of 48–72 h. In animal experiments and clinical trials, MSCs have been reported to be safe and well tolerated [1]. An intriguing feature of these cells is their ability to evade immune recognition due to the lack of expression of HLA class II antigens and the costimulatory molecules CD40, CD80 and CD86. In addition, MSCs have an immunomodulatory function mediated by cell-to-cell contact or by soluble factors when activated by proinflammatory cytokines. This property and the mechanisms involved have been detailed in several previous publications [2–4].

Evidence has also shown that under certain culture conditions, MSCs can differentiate into cells that do not belong to the mesodermal lineage. Successful differentiation of murine bone marrow-derived MSCs into insulin-producing cells (IPCs) was reported as early as 2004 [5–8]. These observations were reproduced by Sun et al., using human bone marrow-derived MSCs (hBM-MSCs) [9]. A final proof of concept was provided by Gabr and associates in 2013 [10]. These researchers differentiated hBM-MSCs into IPCs. Their findings met all the required criteria for the successful production of IPCs. At the end of the differentiation protocol, the percentage of cells testing positive for insulin and c-peptide was modest (~ 5%). The differentiation protocol was later optimized, and the yield increased by threefold to fourfold [11]. Published reports by several investigators support these findings [12–16]. Nevertheless, some important questions need to be addressed: Can these cells retain their immunomodulatory function following differentiation into IPCs? Can they evade and contain allogenic immune responses following their transplantation? Published reports addressing these questions are scanty, controversial and subject to criticism. Wang and associates demonstrated that transplantation of IPCs differentiated from human umbilical cord MSCs could alleviate hyperglycemia in NOD mice [17]. Wu et al. reported that co-transplantation of hBM-MSCs improved the results of islet transplantation in a humanized diabetic mouse model [18]. They attributed this improvement to inhibition of alloreactive T-cells and proliferation of Tregs. On the other hand, Yang et al. reported that IPCs derived from the human umbilical cord were hypoimmunogenic in vitro but evoked an allogenic immune response after their transplantation in immune-competent mice [19]. Hassanin and associates reported that IPCs differentiated from human umbilical cord Wharton’s jelly evoked an immune response following their transplantation into chemically induced diabetic rats [20]. It is surprising to assume that the potential immunomodulation mediated by MSCs can be effective across such a wide xenogeneic spectrum. In an in vitro investigation, Mohammadi et al. noted that IPCs differentiated from murine BM-MSCs acquired immunogenic properties [21]. However, with in vitro experiments, the influence of the in vivo microenvironment is overlooked. A more objective answer can be obtained by the transplantation of human MSC-derived IPCs into humanized mice which is the focus of our current investigation.

## Methods

### Retrieval, expansion and differentiation of human adipose tissue mesenchymal stem cells (hAT-MSCs)

The required approval for this study was obtained from the Ethical Committee of the University of Mansoura (IRB: R. 21.10.1478). Liposuction aspirates were obtained from 3 consenting healthy subjects during elective cosmetic surgeries and their HLA phenotype was determined (Additional file 1: Data S1). The aspirates were digested with 0.075% collagenase type I (Sigma-Aldrich, St. Louis, USA) for 30 min at 37 °C with gentle stirring. Collagenase was inactivated with an equal volume of low-glucose-Dulbecco’s modified Eagle’s complete medium (DMEM-LG, Sigma-Aldrich) and the aspirates were centrifuged for 10 min at 300xg. The cell pellet was resuspended in DMEM-LG supplemented with 10% fetal bovine serum (FBS) and filtered through a 100 µm mesh filter to remove debris. The resuspended cells were plated at a density of 1 × 10^5^/cm^2^ in 75 cm^2^ culture flasks and incubated at 37 °C in a 5% CO_2_ incubator. Three days later, the nonadherent cells were discarded. The adherent cells were cultured to 80% confluence before passaging with a trypsin–EDTA solution. The cells were recultured in complete DMEM-LG, replated at a ratio of 1:2 and cultured to 80% confluence. This step was repeated for three passages. At this point, the cells were spindle-shaped and displayed a fibroblast-like appearance. Their phenotype was determined and their ability to undergo trilineage differentiation into chondrocytes, adipocytes and osteocytes was tested.

At passages 3–5, cultured cells were rinsed with 1× DPBS without Mg^2+^ and Ca^2+^ (Invitrogen, Waltham, Massachusetts, USA) followed by incubation with trypsin–EDTA solution (Gibco, NY, USA) for 3 min, at room temperature. Detached single cells were rinsed with low-glucose (1 g glucose/L) xeno-free human MSC medium (R & D Systems, MU, USA) and centrifuged at 300xg for 5 min. The resulting cell pellet was resuspended in the same medium, seeded in laminin 521-coated flasks at 1 × 10^5^ cells/cm^2^ and cultured for 48 h (Biolamina, Stockholm, Sweden). Subsequently, the cells were cultured in serum-free DMEM-LG supplemented with 100 ng/ml activin-A (R & D Systems), 3 µM CHIR99021 (Sigma-Aldrich), 100 nM of wortmannin (ENZO life Sciences Inc., NY, USA) and 1% B-27 minus insulin (Life Technologies Corporation, NY, USA) for two days. The cells were then cultured in DMEM-LG with 100 ng/ml activin-A, 3 µM CHIR99021 and 1% B-27 minus insulin for two days and then cultured overnight in a serum free DMEM-LG supplemented with 55 nM trichostatin-A (Sigma-Aldrich). For the next 12 days, the cells were cultured in high glucose (4.5 g glucose/L) human MSC-medium supplemented with10 mM nicotinamide (Sigma-Aldrich), 10 nM glucagon-like peptide-1 (Sigma—Aldrich), 10 µg/l PRDX6 protein (Biovision, CA, USA) and 0.1 nM exendin-4 (Sigma-Aldrich). The media were replaced every three days. Finally, the cells were treated with trypsin–EDTA solution. The resulting single cell suspension was seeded in ultralow adherent flasks (Sigma-Aldrich) for three days using the same media. This step allowed the cells to aggregate and form islet-like clusters.

At the end of the differentiation protocol, the cells were evaluated by immunocytochemistry. The proportion of insulin-and c-peptide-positive cells was quantitated by flow cytometry and the expression of the relevant pancreatic endocrine genes was evaluated by RT-PCR.

### The experimental procedure and laboratory methods

Female humanized (NOG-EXL) mice with an HLA-A2-positive phenotype were purchased from Taconic Bioscience (Rensselaer, NY, USA). The animals were housed in individual cages in a sterile room, on 12-h light–dark cycle and provided ad libitum access to food (NIH#31 M rodent diet) and water. The animals were divided into four groups: diabetic animals transplanted with IPCs (no = 16), diabetic animals that were not transplanted (no = 5), normal animals transplanted with IPCs (no = 8) and normal untreated animals (no = 6). Diabetes was chemically induced by intraperitoneal administration of streptozotocin (STZ). Initially, a dose of 160–180 mg/kg was used. This dose was extremely hepatotoxic for NOG-EXL mice ultimately leading to their death. As a result, the dose of STZ was modified to 100–120 mg/kg divided into two equal doses given on two consecutive days. Mice were considered diabetic if their 2-h postprandial blood glucose levels exceeded 200 mg/dL twice.

For transplantation, 3 × 10^6^ of differentiated cells were engrafted under the renal capsule. All animals were followed up weekly to monitor their weight, and 2-h postprandial sugar levels measured by a glucometer (Precichek, Emsdelten, Germany). Serum human and mouse insulin levels were determined by an ELISA kit following the manufacturer’s instructions (Chongqing, Biospes, China, # BEK1242). The observation period ranged from 6 to 8 weeks. Finally, the animals were euthanized. The cell-bearing kidneys and native pancreata were harvested for histology and immunohistochemistry. Blood, spleen and bone marrow samples were also retrieved for immune studies.

#### Gene expression by real-time PCR

Total RNA was extracted from undifferentiated cells and cells at the end of the in vitro differentiation using a Direct-ZolTM RNA Miniprep kit (Zymo Research, California, USA). The RNA concentration was measured with a spectrophotometer (Nanodrop 2000, Thermo Fisher Scientific, Massachusetts, USA). Thereafter, three micrograms of total RNA were converted into cDNA using an RT2 First Strand Kit (Qiagen Sciences, Germantown, MD, USA). Primers were designed using the website of the National Center for Biotechnology Information (Additional file 1: Data S2). In this study, the expression of the relevant pancreatic endocrine genes was evaluated. Expression was determined for the following genes: the pancreatic endocrine hormones insulin (INS), glucagon (GCG) and somatostatin (SST); the relevant transcription factors pancreatic and duodenal homeobox 1 (PDX1), neurogenin3 (NGN3), regulatory factor X6 (RFX6), neurogenic differentiation factor 1 (NEUROD1), v-Maf musculoaponeurotic fibrosarcoma oncogene homologe A and B (MAFA & MAFB) and paired box 4 (PAX4); the pancreatic enzymes: glucokinase (GCK); the glucose transporter solute carrier family member 2 (GLUT-2); the endocrine precursor marker nestin (NES); and the nuclear hormone receptor superfamily member estrogen-related receptor gamma (ESRRγ). Glyceraldehyde-3-phosphate dehydrogenase (GAPDH) was included as an internal control and for normalization. Amplification was performed for each sample in a 20 µL reaction volume consisting of 10 µL of 2X Maxima SYBR Green Master Mix (Thermo Fisher Scientific), 2 µL of primers (5 nmol), 1 µL of cDNA template (100 nmol) and 7 µL of nuclease-free water. The reactions were carried out in a 96-well plate inserted into a real-time thermal cycler (CFX96 Real-Time System, Bio-Rad, Hercules, CA, USA). The cycling parameters for PCR amplification were programmed as follows: initial denaturation at 95 °C for 3 min, followed by 40 cycles of denaturation at 95 °C for 15 s, annealing at 60 °C for 30 s and extension at 72 °C for 30 s. The procedure was performed in triplicate for each sample. A mathematical model introduced by Pfaffl [22] was used to calculate the relative expression of the target genes. In this study, the data are expressed relative to those obtained for undifferentiated MSCs.

#### Immunolabeling

*For immunocytochemistry and* immunohistochemistry*:* the primary antibodies used were mouse monoclonal anti-insulin (#8138, Cell Signaling Technology, Danvers, MA, USA), rabbit monoclonal anti-glucagon (# 8233, Cell Signaling Technology), rabbit polyclonal anti-c-peptide (# 4593, Cell Signaling Technology) and rabbit polyclonal anti-human somatostatin (# GTX39061, Gene Tex, Alton Pkwy Irvine, CA, USA). The secondary antibodies used were (H + L Alexa flour 488 conjugate) anti-mouse IgG heavy and light (# 4408, Cell Signaling Technology), and Alexa flour 555 conjugate anti-rabbit IgG (H + L) (# 4413, Cell Signaling Technology). The nuclei were counterstained with DAPI (# 4083, Cell Signaling Technology). *For intracellular staining for flow cytometry*: Primary monoclonal antibodies against insulin (# 565688, BD, San Jose, CA, USA), and c-peptide (# 565830, BD) and for secondary antibody anti mouse IgG Fab2 Alexa Flour 488 (# 8878 Cell Signaling Technology) were used. *For surface marker staining of immune cell subsets by flow cytometry,* the following conjugated antibodies were used: CD45 phycoerythrin Cy7 (PE Cy7) (# 557748, BD), CD19 phycoerythrin CF594 (PE CF594) (# 562294, BD), CD3 fluorescein isothiocyanate (FITC) (# 345763, BD), CD4 allophycocyanin (APC) (# 345771, BD), CD8 phycoerythrin (PE) (# 555367, BD), CD69 (FITC) (# 347823, BD), CD16 allophycocyanin H7 (APC H7) (# 560195, BD), human leucocytic antigen (HLA)-ABC (FITC) (# 557348, BD) and HLA-DR (APC) (# 347403, BD). *For immunohistochemical staining of CD45*^+^ *cells, an* antihuman antibody against CD45^+^ was used (Clones 2B11 + PD7/26, Dako, Glostrup, Denmark). For immunohistochemistry of the native pancreata, the primary antibody used was mouse monoclonal anti-insulin (# Ab20756, Abcam Cambridge, UK), and the secondary antibody was the power-stain 1.0 poly-HRP DAB Kit for mouse (# 0205H, BioSB, Santa Barbara, CA, USA). The sections were examined by light microscopy (Olympus, BX51, Tokyo, Japan).

#### Immunocytochemistry of differentiated cells

The cells were plated on chamber slides (Nunc, Rochester, NY; USA), and fixed with 4% paraformaldehyde for 10 min at room temperature. The cells were then permeabilized with 100% chilled ethanol for 10 min, blocked with 5% normal goat serum for 60 min and incubated overnight with the primary antibodies. Subsequently, the cells were washed with PBS and incubated with the secondary antibodies for 2 h at RT. Nuclei were counterstained with DAPI. Negative controls were prepared by omitting the primary antibody and by staining of undifferentiated cells. Confocal images were captured using a Leica TCS SP8 microscope (Leica microsystems, Mannheim, Germany).

#### Quantitation of cells positive for insulin and c-peptide by flow-cytometry

The protocols used for cell preparation and labeling are detailed in (Additional file 1: Data S3). Labeled cells were identified using a red laser with a wavelength of 488 by the FACS ARIA III cell sorter (Becton, Dickinson). A total of 20,000 events were obtained. Stained and unstained undifferentiated cells and differentiated cells processed without the primary antibody served as negative controls. The data were analyzed by Flow Jo software (Becton, Dickinson).

#### Immunohistochemistry of the cell-bearing kidneys

The harvested kidneys were formalin fixed, paraffin embedded and sectioned at 3 µm. The sections were plated on coated positively charged slides (Citotest., Haimen, China). The sections were deparaffinized using xylene and rehydrated using a decreasing ethanol gradient. The antigens were unmasked by boiling the slides in 10 mM sodium citrate buffer (pH 6.0) and subsequently incubating them at sub-boiling temperature for 10 min. The sections were blocked with 5% normal goat serum and then incubated overnight with the primary antibody at 4 °C. The slides were washed three times in PBS and incubated with the secondary antibody for 2 h at RT. The nuclei were counterstained using DAPI. A kidney obtained from a normal animal was also stained to serve as a control. Confocal images were captured using a Leica TCS SP8 microscope (Leica Microsystems, Mannheim, Germany).

#### Immunohistochemical staining of cell-bearing kidneys for CD45^+^ cells

Consecutive paraffin sections (3 μm) from each kidney sample were obtained. Antigen unmasking was carried out by heat-induced antigen retrieval using buffered sodium citrate according to the manufacturer’s instructions (Dako). The antibody against CD45 was then applied. Human lymph node sections were used as a positive control. Negative controls were obtained by omitting the primary antibody. Immunohistochemical reactions were performed using an automated immunohistochemical stainer (Autostainer Link 48, Dako). The primary antibody was visualized using Dako Envision and diaminobenzidine (DAB)-based substrate. The sections were counterstained with hematoxylin and examined with a light microscope (Olympus BX51, Tokyo, Japan).

#### Quantitation of immune cell subsets by flowcytometry

**Fig. 1:**
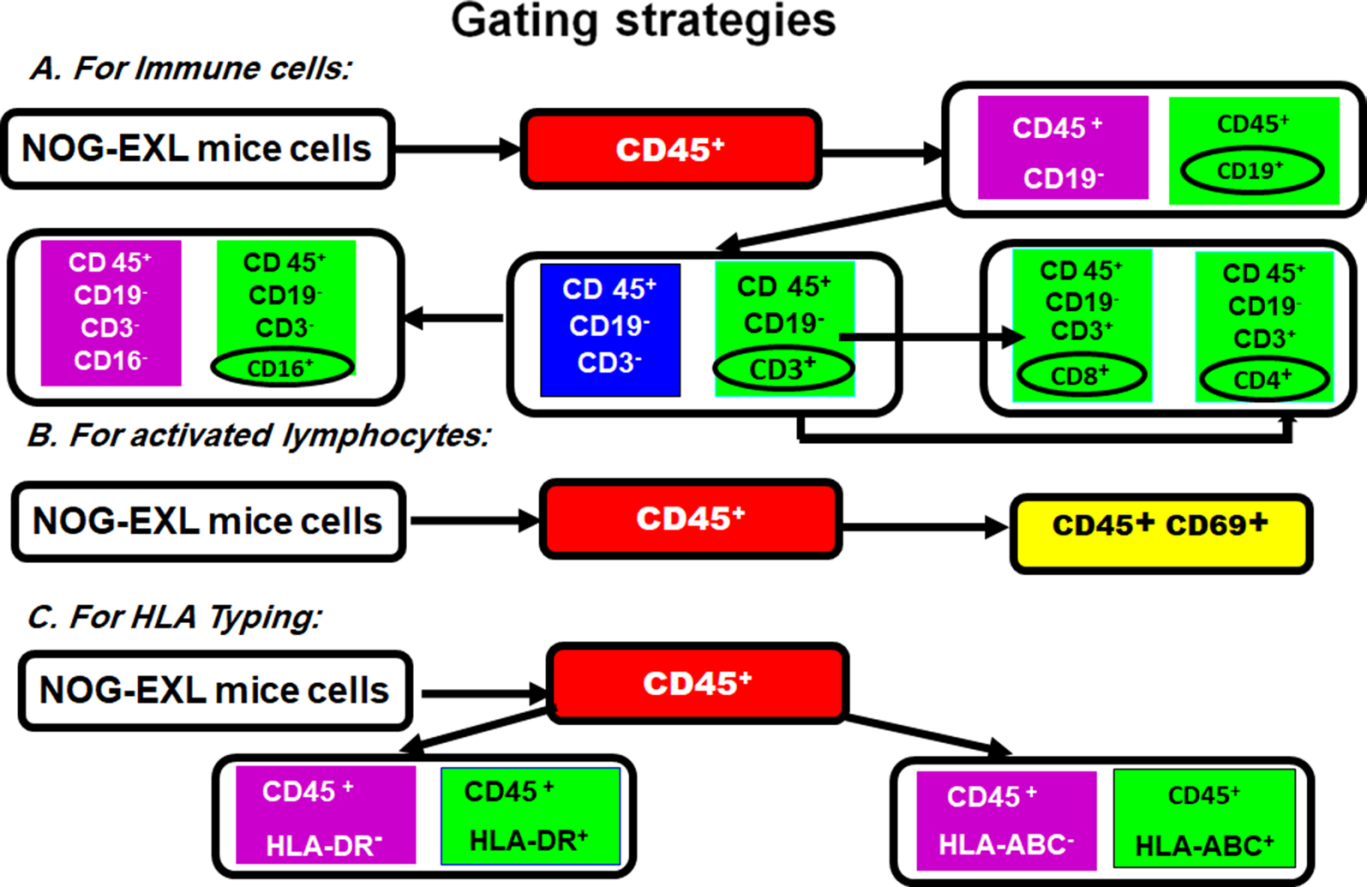
The gating strategy for the study of immune cell subsets by flow cytometry. Initially, CD45^+^ cells were identified. Then, CD19^+^ cells were sorted out. Subsequently CD3^+^, CD4^+^ and CD8^+^ cells were sorted out from CD45^+^ and CD19^−^ cells. CD16^+^ cells were then sorted out from CD45^+^, CD19^−^, CD^−^, CD3^−^ cells. Activated lymphocytes (CD69^+^) were identified within the CD45^+^ cell-population. HLA-ABC^+^ and HLA-DR^+^ cells were also identified among the CD45^+^ cell-population

The protocols used to prepare cell samples from the blood, spleens and bone marrow of NOG-EXL mice are detailed in Additional file 1: Data S4. Aliquots (100 µL) of the prepared cell suspension with a concentration of 1 × 10^6^ cells were stained for the different surface markers by adding 3 µL of conjugated antibody and incubating the cells at 4 °C for 30 min in the dark. Then, the cells were washed with 500 µL of staining buffer and centrifuged at 600×*g* for 5 min. The supernatant was discarded, and cells were resuspended in 500 µL of staining buffer. The labeled cells were identified by a red laser with a wavelength of 488 and a blue laser with a wavelength of 633 using a FACS ARIA III cell sorter (Becton, Dickinson). A total of 20,000 events were obtained and analyzed by FACS DIVA software (Becton, Dickinson). The gating strategy is outlined in Fig. 1.

### Statistical analysis

Data analysis was carried out using IBM SPSS statistics 16.0 software (IBM Corp., Armonk, NY, USA). According to their category, data were expressed as median values or as a mean ± standard error. Since the data were nonparametric and unmatched, statistical differences between 2 groups were analyzed by the Mann–Whitney test. For more than 2 groups, Kruskal—Wallis 1-way analysis of variance was used. A *p value* of < 0.05 was considered significant.

## Results

### Identity of the AT-MSCs

AT-MSCs were retrieved from 3 HLA-A2-negative donors. At the end of the expansion protocol, the cells adhered to the plastic and exhibited a spindle shaped morphology. The cells were positive for the MSC surface markers: CD73, CD90 and CD105 and were negative for the hematopoietic stem cell markers: CD14, CD34 and CD45 (Additional file 1: Data S5). In addition, the cells could be differentiated into adipocytes, chondrocytes and osteocytes when the appropriate growth factors were used (Additional file 1: Data S6).

### Characterization of the differentiated cells

#### Immunocytochemistry

**Fig. 2:**
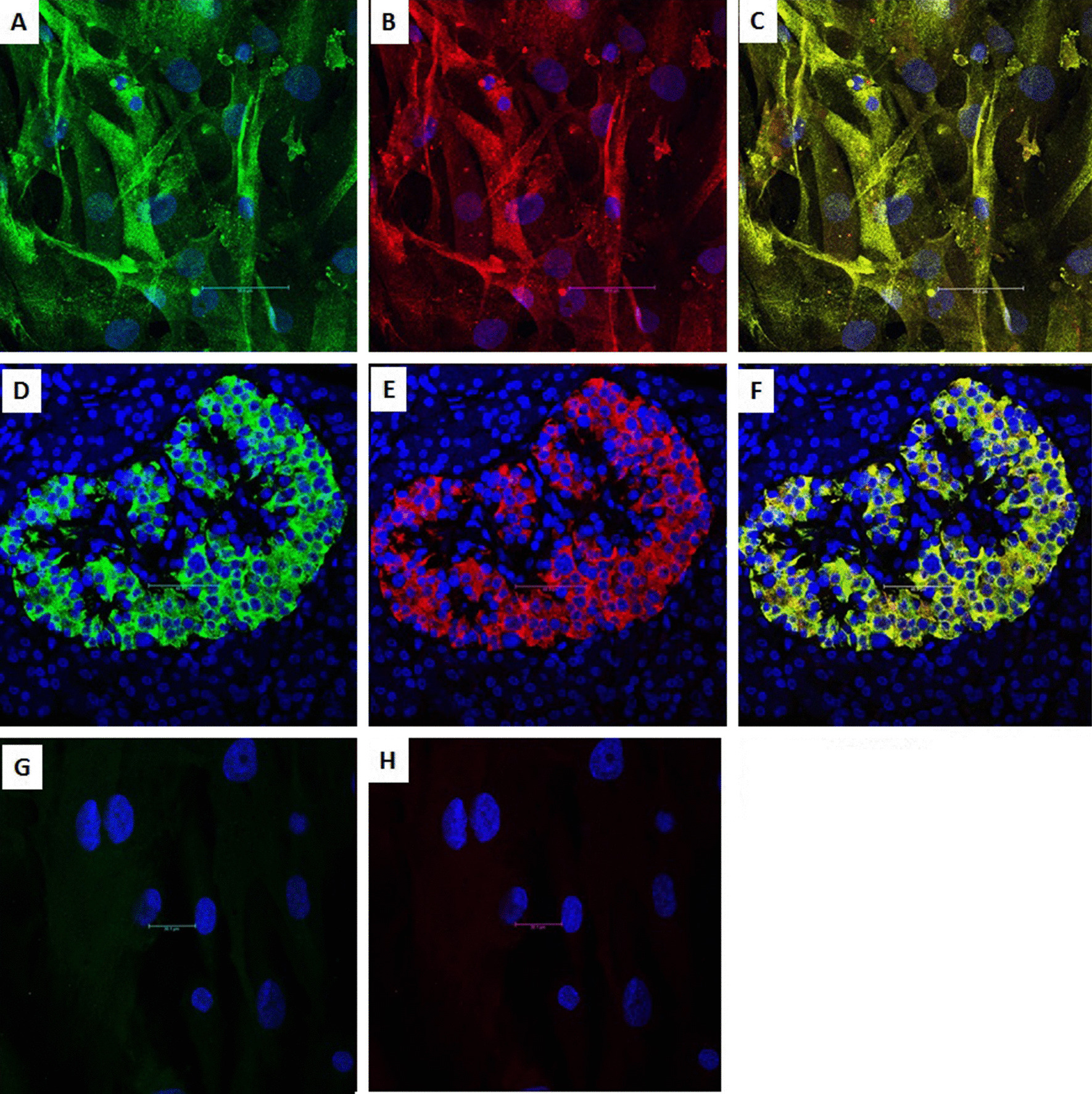
Immunocytochemical staining of differentiated MSCs. **A** Positive staining for insulin (green). **B** Positive staining for c-peptide (red). **C** Merged image of insulin and c-peptide (yellow) showing that insulin and c-peptide were coexpressed within the same cells. Nuclei were stained with DAPI (blue). Immunohistology of a human islet (positive control): **D** positive for insulin (green), **E** positive for c-peptide (red) and **F** merged image of insulin and c-peptide (yellow). Immunocytochemical staining of undifferentiated MSCs (negative control): **G** Negative for insulin. **H** Negative for c-peptide

Immunofluorescence demonstrated that the differentiated cells were positive for insulin and c-peptide. Furthermore, insulin and c-peptide were coexpressed within the same cells (Fig. 2). A positive control was obtained by staining of human islets. A negative control was obtained by staining undifferentiated cells.

#### Quantitation of the hormone-positive cells by flow-cytometry

**Fig. 3:**
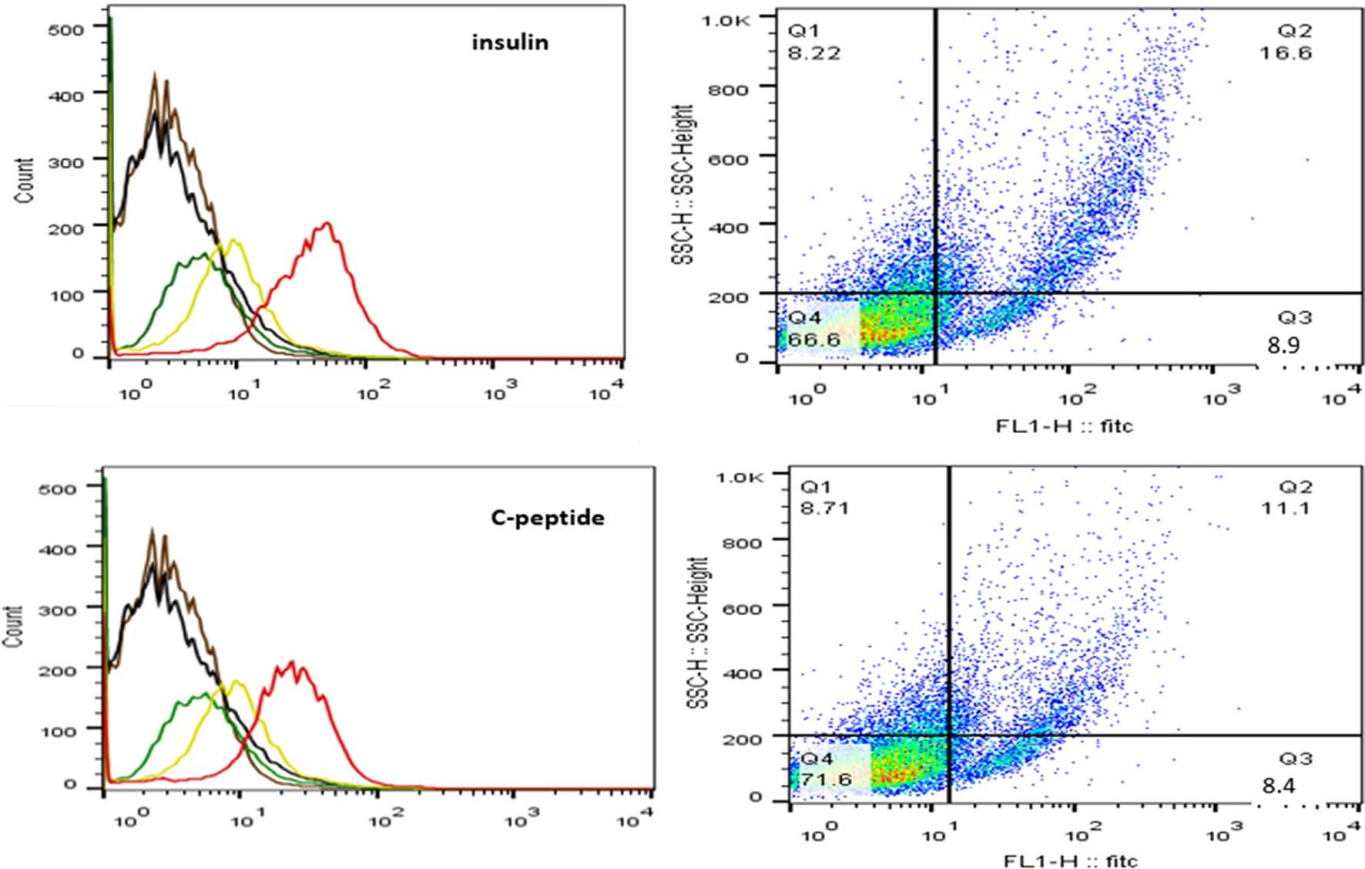
Determination of insulin-and c-peptide-positive cells by flow cytometry; a representative example. Undifferentiated unstained cells (black), undifferentiated stained cells (orange), differentiated unstained cells (green) and differentiated cells stained with omission of the primary antibody (yellow) served as negative controls. Differentiated cells stained with the primary and secondary antibodies (red). The proportion of insulin-positive cells was 25.5% and c-peptide-positive cells was 19.5%

The proportion of insulin-positive cells ranged from 18–22% and c-peptide-positive cells ranged from 16–20%. A representative example is shown in (Fig. 3).

#### *RT-PCR* (Fig. 4)

**Fig. 4:**
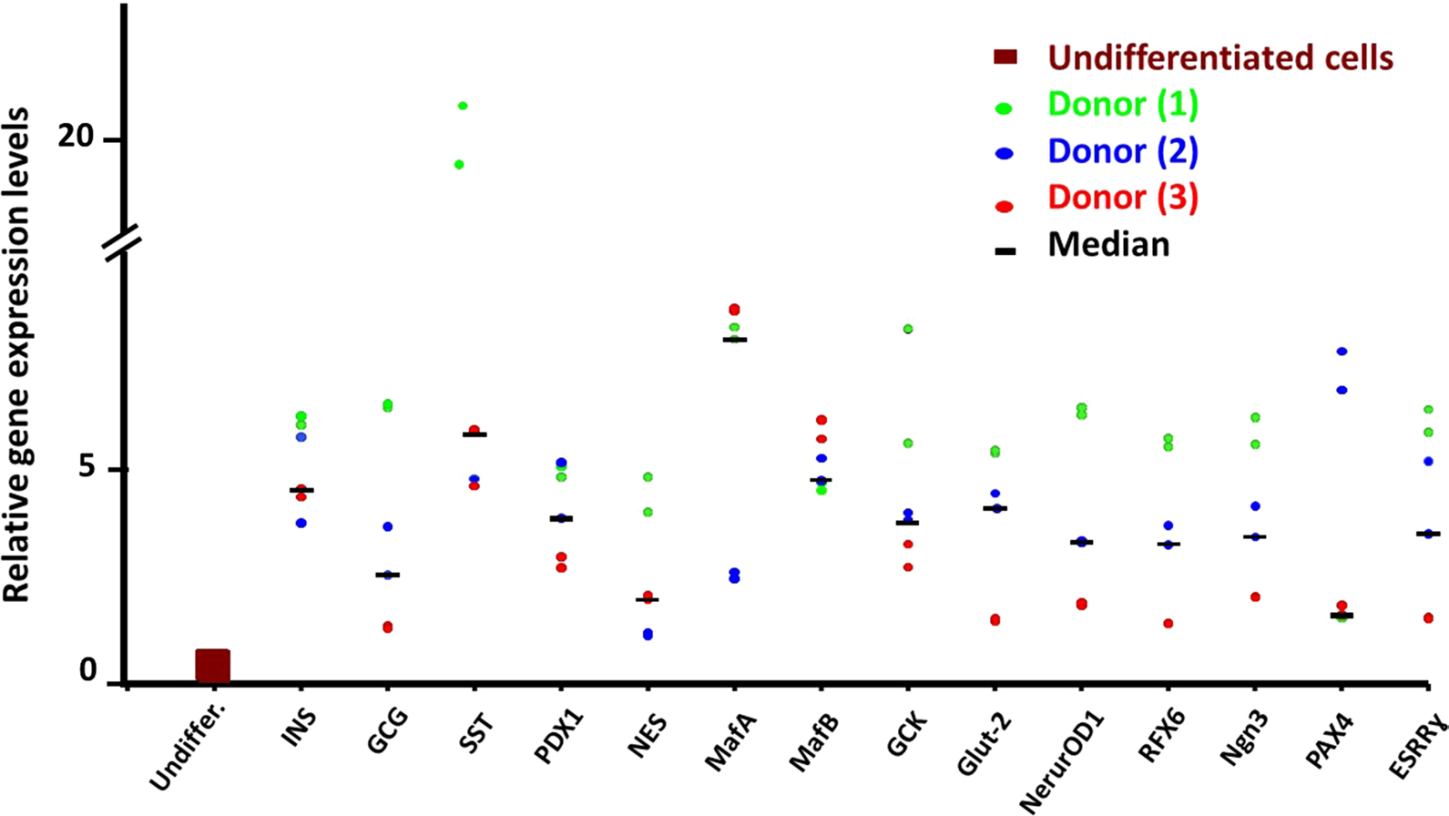
RT-PCR: Relative gene expression of differentiated cells derived from 3 donors. At the end of the differentiation protocol, all the relevant pancreatic endocrine genes were expressed. These included genes encoding endocrine hormones *(INS, GCG, SST),* genes encoding transcription factors *(PDX1, NES, MAFA, MAFB, NEUROD1, RFX6, NGN3, PAX4, ESR*R ɣ) and genes encoding pancreatic enzymes *(GCK, GLUT-2).* Transverse bars represent median values

*RT-PCR was performed* at the end of the differentiation protocol. The results revealed that all the relevant pancreatic endocrine genes were expressed. These included genes encoding endocrine hormones *(INS, GCG, SST)*, genes encoding transcription factors *(PDX1, NES, MAFA, MAFB NEUROD1, RFX6, NGN3, PAX4, ESRR ɣ)* and genes encoding the pancreatic enzyme *(GCK) and the glucose transporter GLUT-2).* (Raw data, Additional file 1: Data S7).

#### Outcomes of the in-vivo transplantation (*Fig. **5*)

**Fig. 5:**
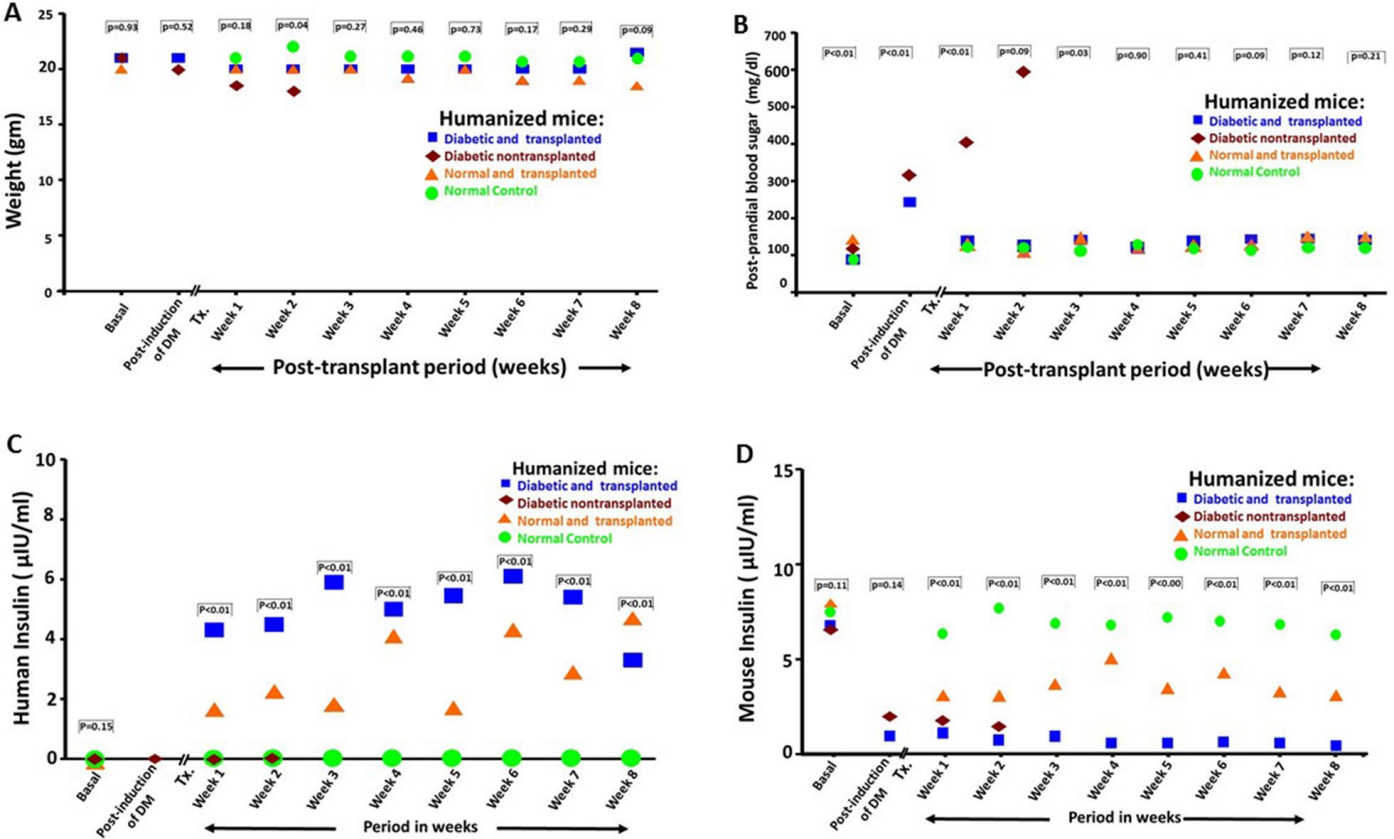
Follow-up evaluation. Data are expressed as median values. Diabetic and transplanted animals (blue squares). Diabetic untransplanted animals (brown) diamonds). Normal and transplanted animals (orange triangles). Normal untreated controls (green circles). **A** Weight: There was a progressive loss of weight among the diabetic untransplanted animals until time of death. The weights of the other 3 groups were comparable throughout the observation period. **B** Glucose levels: After induction of diabetes, the blood glucose levels increased sharply and remained high among the nontransplanted mice while they were normalized within 2 weeks in the transplanted mice; thereafter, euglycemia was maintained and similar to those of controls. **C** Human insulin: Human insulin became measurable among the diabetic transplanted mice as well as the nondiabetic transplanted ones. It was not measurable among the diabetic untransplanted animals or the normal treated controls. **D** Mouse insulin: Following STZ-induction, mouse insulin levels decreased sharply among the diabetic transplanted or untransplanted animals. They remained measurable among the nondiabetic transplanted animals as well as the normal controls

Out of the diabetic and transplanted group, only 9 mice completed their follow-up. Four animals died from STZ-induced toxic hepatitis. One animal died during surgery and another two shortly thereafter. Body weight remained stable and comparable among 3 groups of animals: the normal controls, the normal transplanted and the diabetic transplanted mice. STZ administration resulted in a sharp increase in the blood sugar levels and a significant decrease in the mouse insulin to negligible levels. Following cell transplantation, the blood glucose levels of the diabetic animals decreased to euglycemic levels within 2 weeks. Furthermore, human insulin levels became measurable throughout the observation period. Human insulin and mouse insulin levels were quantifiable among the normal nondiabetic animals that underwent transplantation. However, the human insulin values of these animals were lower than those of the diabetic animals that underwent transplantation, and their mouse insulin levels were lower than those of the normal control animals. Diabetic but nontransplanted animals remained hyperglycemic with progressive weight loss and died 10–14 days after induction of diabetes. (Raw data, Additional file 1: Data S8).

#### Immunohistochemistry of the cell-bearing kidneys and the native pancreata

**Fig. 6:**
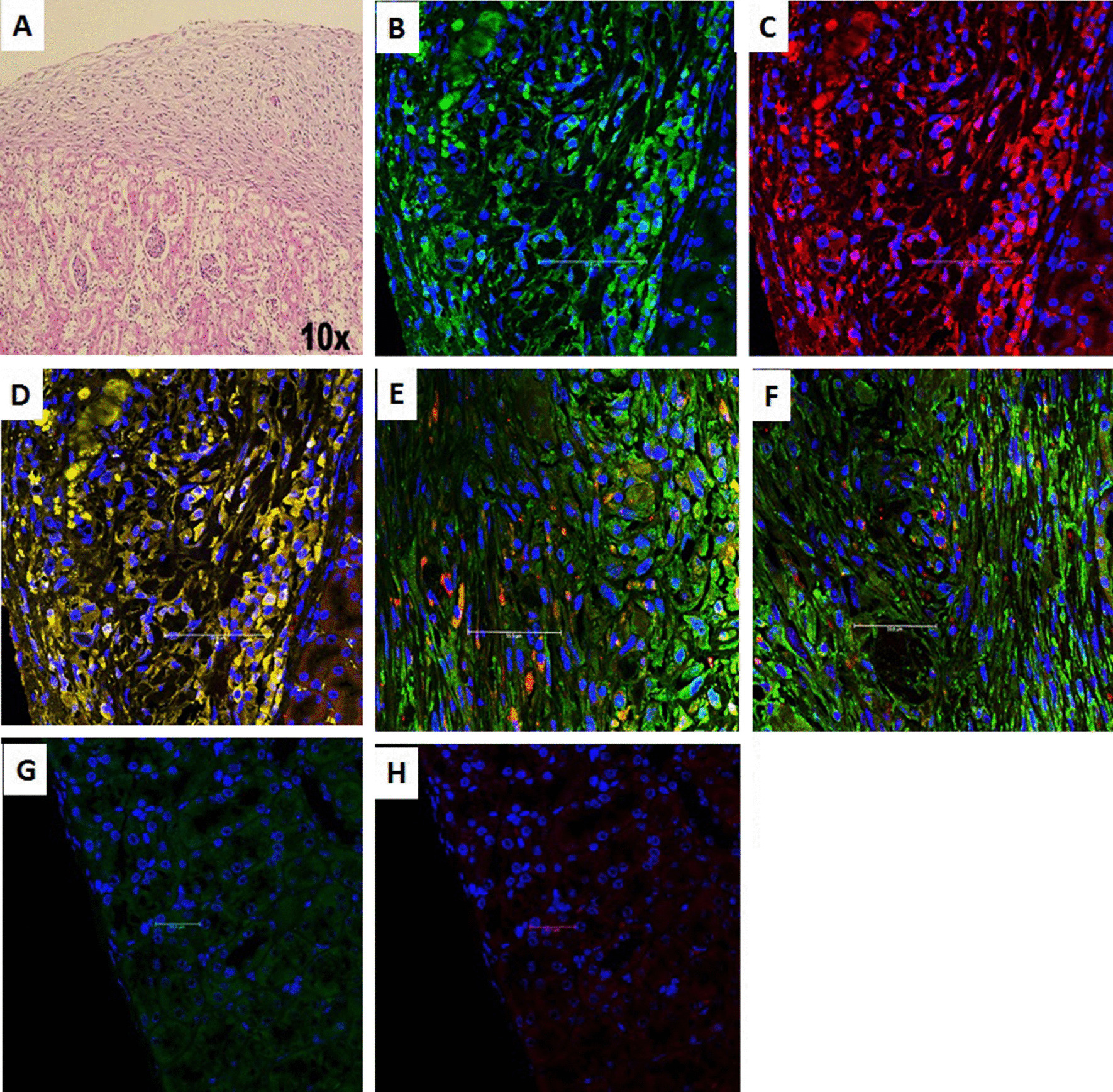
Histology and immunohistochemistry of an explanted cell-bearing kidney from a diabetic transplanted animal. **A** Hematoxylin and eosin staining showing transplanted cells under the renal capsule. **B** Insulin-positive cells (green). **C** C-peptide-positive cells (red). **D** Coexpression of insulin and c-peptide within the same cells (yellow). **E** Glucagon-positive cells (red) were in a different cell population than insulin-positive cells. **F** Somatostatin-positive cells (red) were in a different cell population than insulin-positive cells. Control staining of a kidney explanted from a normal control. **G** Negative for insulin. **H** Negative for c-peptide

**Fig. 7:**
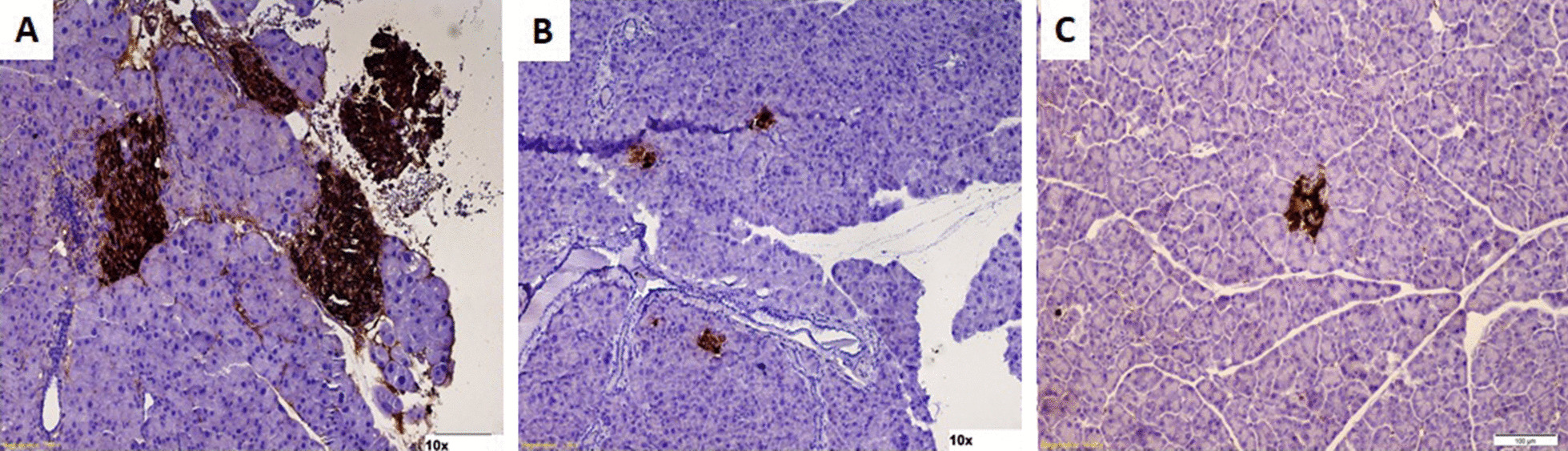
Immunohistochemistry of a harvested pancreas. **A** Pancreas from a normal untreated animal showing distinct well-stained islets **B.** Pancreas from STZ-induced diabetic mouse that underwent transplantation showing very few atrophic islets. **C** Pancreas from a diabetic nontransplanted mouse, showing very few atrophic islets

**Fig. 8:**
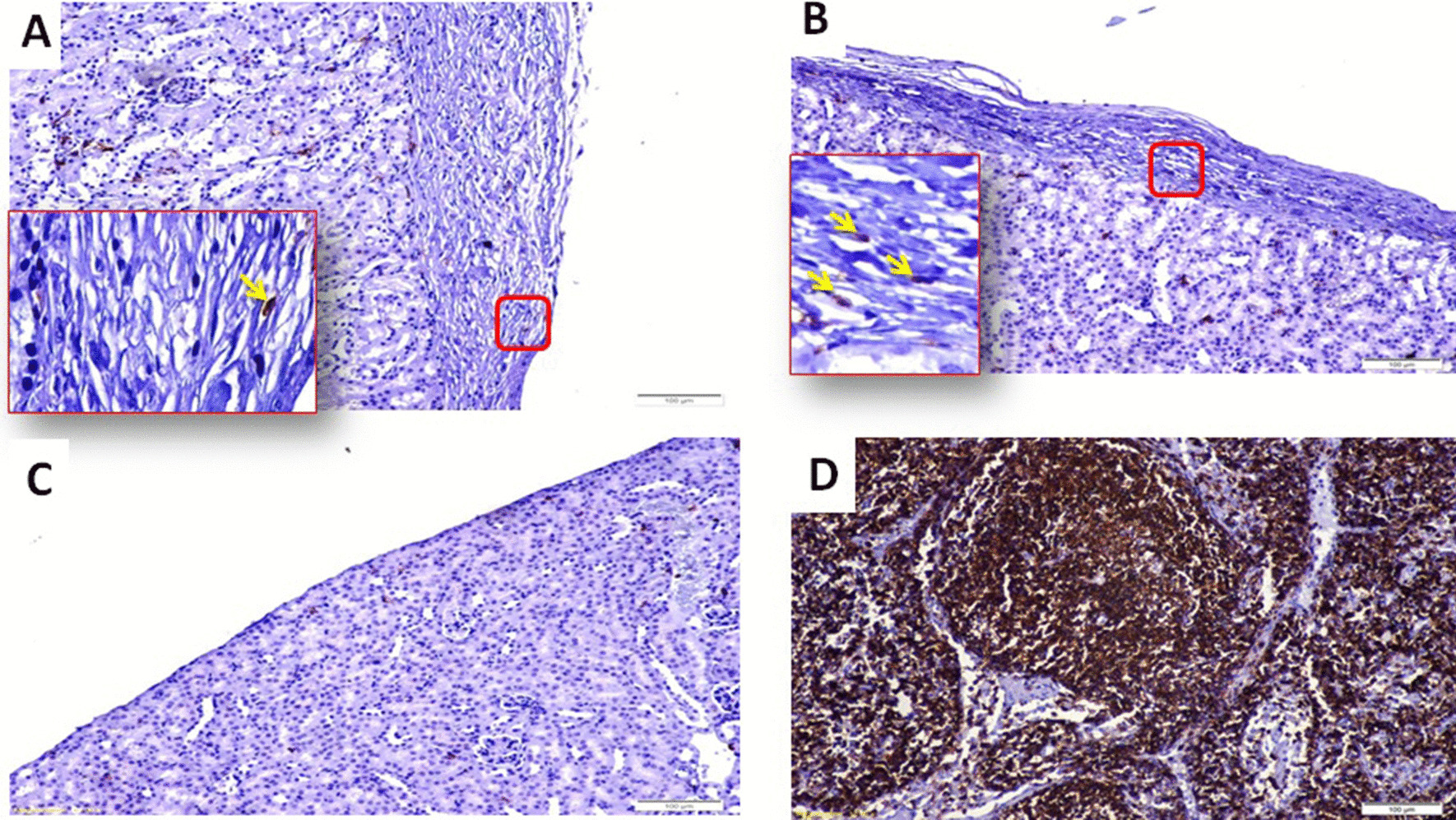
CD45^+^ staining of kidneys from transplanted mice: **A** Immunostaining of IPCs transplanted under the renal capsule of a diabetic humanized mouse with anti-CD45^+^ antibody. There were very few CD45^+^ cells. The yellow arrow inside the high magnification inset points to a CD45^+^ cell (brown). **B** Immunostaining of IPCs transplanted under the renal capsule of a normal nondiabetic animal with anti-CD45 antibody. There were few CD45^+^ cells. The yellow arrows inside the high magnification inset point to CD45^+^ cells (brown). **C** Immunostaining of a cross section of a kidney from a normal animal that was not transplanted. Immunostaining with an anti-CD45 antibody showing the absence of CD45^+^ cells. **D** Positive control: Cross section of a lymph node. Immunostaining with an anti-CD45 antibody showed a large number of CD45^+^ cells (brown)

Immunofluorescence showed that the transplanted cells under the renal capsule of diabetic mice were insulin-and c-peptide-positive and that these two molecules were coexpressed within the same cells. Glucagon- and somatostatin-positive cells were also observed but localized in a different cell population (Fig. 6). A negative control was obtained by staining of a kidney harvested from a normal untreated mouse. Examination of the native pancreata of diabetic mice that underwent transplantation revealed very few atrophic islets compared to the normal control group (Fig. 7). The presence of CD45^+^ cells was sparse among the transplanted IPCs under the kidney capsule in diabetic and nondiabetic animals (Fig. 8).

#### Quantitation of immune cell subsets by flow cytometry

**Fig. 9:**
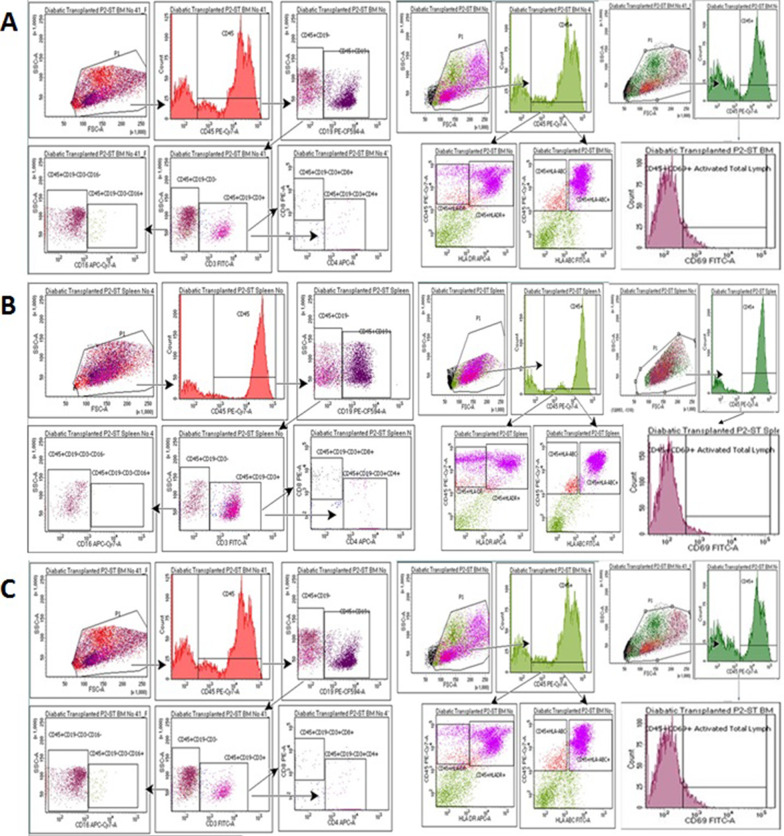
The results of flow cytometry for immune cell subsets from samples obtained from a representative example of a diabetic humanized mouse that underwent transplantation: **A** Blood sample: CD19^+^ cells accounted for 35% and CD69^+^ for 0.7% of the CD45^+^ cells. CD4^+^ cells accounted for 57% and CD8^+^ for 34% of the CD3^+^ population. The proportion of CD45^+^, CD19^−^, CD3^−^ and CD16^+^ cells was 6.6%. The proportion of cells expressing HLA-ABC and of HLA-DR was 98% and 42%, respectively. **B** Splenocytes: CD19^+^ cells accounted for 57% and CD69^+^ cells for 5.5% of CD45^+^ cells. The CD3^+^ cells and CD4^+^ cells accounted for 51% and CD8^+^ cells for 41% of the CD3^+^ cells. The proportion of CD45^+^, CD19^−^, CD3^−^ and CD16^+^ cells was 1.8%. The proportion of cells expressing HLA-ABC and of HLA-DR were 87% and 49%, respectively. **C** Bone marrow: CD19^+^ cells accounted for 51% and CD69^+^ cells for 11.4% of the CD45^+^ cells. CD4^+^ cells accounted for 73% and CD8 + cells for 17% of the CD3^+^ cell population. The proportion of CD45^+^, CD19^−^, CD3^−^ and CD16^+^ cells was 3.8%. The proportion of cells expressing HLA-ABC and of HLA-DR was 82% and 74%, respectively

**Fig. 10:**
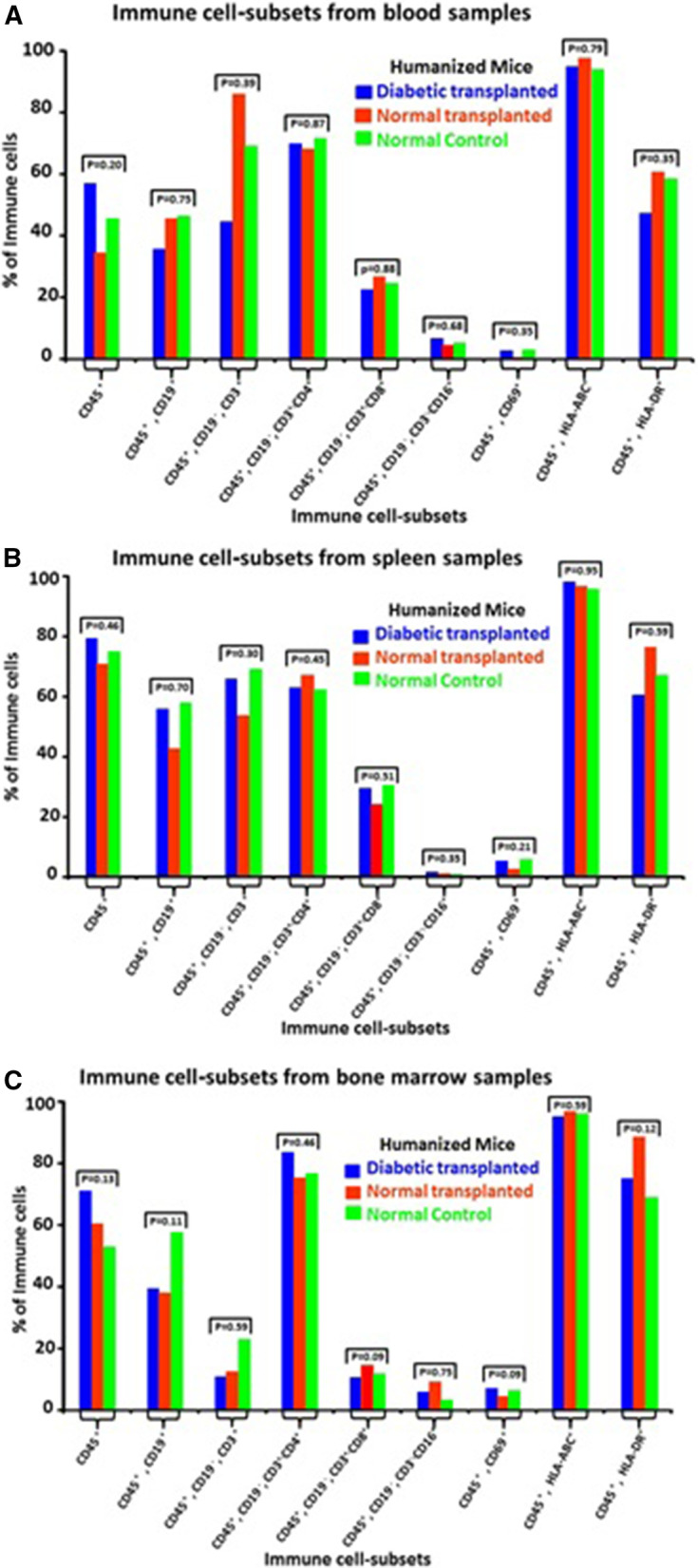
Comparisons of the proportions of immune cell subsets in blood, spleen and bone marrow samples. Data are expressed as median values. The results revealed that the proportions of immune cell subsets were comparable among the 3 groups of mice without a significant difference. The HLA-ABC and HLA-DR levels were also similar. **A** The proportion of immune cell subsets in blood samples. **B** Immune cell subsets in spleen samples. **C** The proportion of immune cell subsets in bone marrow samples

The results of flow cytometry from representative samples obtained from a diabetic mouse that underwent transplantation are shown in (Fig. 9). Comparisons of the proportions of immune cell subsets obtained from the diabetic transplanted, nondiabetic transplanted and normal mice were comparable and not significantly different (Fig. 10). The studied immune cell subsets included: CD3^+^, CD4^+^, CD8^+^, CD16^+^, CD19^+^, CD69^+^. In addition, the HLA-ABC and HLA-DR expression levels were similar*.* (Raw data, Additional file 1: Data S9).

## Discussion

To the best of our knowledge, this is the first study in which allogenic hMSC-derived IPCs were transplanted into humanized mice to study possible immune responses. In the current study, NOG-EXL mice were used. They represent the second generation of NOG mice which are an inbred strain of NOD mice with a spontaneous mutation of the protein kinase DNA activated, catalytic subunit (*Prkdc*^*scid*^) gene and targeted mutation of the interleukin-2 receptor gamma chain gene (*NOD, Prkdc*^*scid*^*, IL-2rg*^*tm*^) [23]. *IL-2rg* mutation leads to severe impairment of B and T-cell functions and prevents the development of natural killer (NK) cells. Humanization of these mice involves the engraftment of human hematopoietic stem cells (HSCs) obtained from the umbilical cord into juvenile NOG mice. However, following humanization, certain cell lineages fail to develop because of species-specific differences in cytokines. NOG-EXL mice developed by Ito et al. [24] represent a further improvement in which NOG mice bear human genes for the granulocyte macrophage-colony stimulating factor (GM-CSF) and IL-3. Following their transplantation with human HSCs, hNOG-EXL mice have a higher overall engraftment and markedly better development of myeloid cells than hNOG mice. These mice do not spontaneously develop diabetes. For this reason, diabetes was chemically induced by STZ administration. There are several drawbacks to the use of STZ. STZ can vary depending on the used strains of animals or their gender [25]. It can be toxic to the treated animals, transplanted cells and/or the engrafted immune cells. Furthermore, the native islets may recover with time [26]. The modified regimen for STZ-induction of diabetes, utilized in this study, was well tolerated, proved to be safe and effective in inducing hyperglycemia. At the end of the observation period, the pancreata of the STZ-treated animals were harvested and histologically examined to rule out the possibility of spontaneous recovery. Very few atrophic islets were observed. These did not contribute to euglycemia among the transplanted animals since diabetic nontransplanted mice remained hyperglycemic until their ultimate early death. The integrity of the engrafted immune cells was also confirmed by comparing data from the treated animals with those from normal controls.

We opted to use hAT-MSCs rather than hBM-MSCs for differentiation into IPCs for several reasons. In a previous investigation, it was shown that the differentiation rates of hBM-MSCs and hAT-MSCs were similar [27]. Sterm and associates reported that 1 mL of bone marrow aspirate yields 600–1000 BM-MSCs, while an equivalent volume of adipose tissue aspirates provides ~ 5000 AT-MSCs [28]. Ribeiro et al. also noted that AT-MSCs exert a stronger inhibitory effect on T-cell proliferation than BM-MSCs [29]. In addition, large volumes of adipose tissue aspirates are readily available from cosmetic surgical procedures and should not be wasted. Successful differentiation was verified by immunocytochemistry, flow cytometry and RT-PCR. Immunocytochemistry demonstrated that the differentiated cells expressed cytoplasmic insulin and c-peptide positive granules. Flow cytometry showed that 12–18% of cells were c-peptide positive. We believe that c-peptide-based measurements are more accurate than insulin-based ones. Insulin present in the culture media can be absorbed by and sequestered in the cells resulting in false higher readings [30]. According to RT-PCR, all the relevant pancreatic endocrine genes were expressed. Notably, the estrogen-related receptor gamma *(ESRR*_*ɣ*_*)* gene was also expressed. This gene is required for metabolic maturation of the glucose-responsive β-cells [31].

In this study, cells derived from HLA-A2^_^ negative donors were transplanted into HLA-A2 positive^_^humanized mice. Matching at the HLA-A2 locus was avoided and transplantation of allogeneic cells was ensured. The observation period of this study was relatively short. The mice were received from the supplier after human immune cell chimerism was established. As a result, the study window was brief in view of the limited life span of these animals. Nevertheless, an observation period of 6–8 weeks is adequate for the development of allogenic immune responses [32]. Throughout the follow-up period, the animals weight, blood sugar levels and serum levels of human and/or mouse insulin were measured. Blood glucose levels were measured after a 2-h day fast. According to Sun et al., such measurements reflect the metabolic processes of gluconeogenesis and glycogenolysis [33]. Following STZ induction, the blood glucose levels increased to a mean of 236 + 2.2 mg/dL among the diabetic animals. After cell transplantation, euglycemia was achieved within 2 weeks. Serum mouse insulin levels were negligible and human insulin levels became measurable throughout the observation period. The diabetic animals that were not transplanted remained hyperglycemic, again with negligible levels of serum mouse insulin and progressive weight loss. They died 10–14 days after induction of diabetes; a period of time that corresponds to the cure of the diabetic and transplanted mice. This clearly demonstrates that the diabetic model was effective and ensured that euglycemia was the result of cell transplantation. The group of normal and transplanted animals provided a control for the possible impact of the diabetic environment on the immune responses. The level of human insulin in these mice was also quantifiable but lower than those of the normal mice. Moreover, their mouse insulin levels were also lower than those of normal controls. This finding can be explained by the presence of 2 sources of insulin in the transplanted nondiabetic mice: human insulin from the engrafted cells and mouse insulin from their native pancreata.

Immunohistochemistry of the explanted kidneys revealed positive staining for insulin and c-peptide and the coexpression of these two molecules within the same engrafted cells. Glucagon and somatostatin staining was also observed, but these hormones were localized in a different cell population. The appearance of alpha and delta cells reflects further maturation of the transplanted cells under the influence of the favorable in vivo environment [34].

Allogenic immune responses were evaluated by flow cytometry to determine the proportions of immune cell subsets in blood, spleen, and bone marrow samples from the diabetic mice that underwent transplantation, normal animals that were transplanted with IPCs and normal untreated mice. There were no differences in the proportion of cells positive for the common leucocyte antigen (CD45^+^) or the early T-cell activation marker (CD69^+^). The differences in the proportions of innate immune cells (CD16^+^) or adaptive immune cells (CD4^+^, CD8^+^, CD19^+^) were statistically insignificant. Notably, CD16^+^ is also moderately expressed on monocyte subsets and dendritic cells [35]. The CD4^+^/CD8^+^ ratio was greater than 2.5 in all tested specimens which is considered as a normal value [36]. Immune responses were also studied in the cell-bearing kidneys and the engrafted cells by immunohistochemistry. There were few hCD45^+^ cells (a maximum of 2–3 cells/field). As a result, further studies of the CD45^+^ cell subpopulations were judged to be unwarranted.

Collectively, the results of this study confirm that transplantation of allogenic hAT-MSCs into diabetic humanized mice, normalized their blood sugar levels. An allogenic immune response was not detected. Differentiated IPCs accounted for only ≃ 20% of the transplanted cells. It can be postulated that the undifferentiated population exerted an immunomodulatory effect. This suggestion conforms with the findings of Wu et al., who reported that third-party undifferentiated MSCs improved the results of islet transplantation in humanized diabetic mice [18]. Alternatively, the results may indicate a muted immune response by the utilized strain of humanized mice.

## Conclusions

Ideally, cell transplantation for the treatment of insulin-dependent diabetes should not require immunosuppression, encapsulation or genetic manipulation. Immunosuppression has important side effects and some of its agents are diabetogenic. The lack of early oxygenation, poor vascularization and the development of pericapsular fibrosis are some of the problems related to encapsulation. Gene manipulation is a promising strategy, but the potential shortcomings are not yet known. The findings of this study can provide a hope that these issues can be circumvented. The immunomodulatory functions of hAT-MSC-derived IPCs can be further enhanced by several means to overcome allogenic immune responses [1, 37]. Additional issues should also be addressed. The duration for which these cells can maintain their function, the required number of cells and the optimal site for their implantation have also to be studied. Finally, it must be emphasized that cell therapy for type 1DM can only be meaningful and clinically justifiable if functional outcome is better than that of the ever-improving closed-loop insulin pumps.

## Supplementary Information

**Additional file 1**. **Data S1**: Donor HLA typing. **Data S2**: List of human gene-specific primers used in real time PCR. **Data S3**: Quantitation of Insulin- and c-peptide-positive cells by flow cytometry. **Data S4**: Preparation of samples for quantitation of immune cells by flow cytometry. **Data S5**: Immunophenotyping of donor cells. **Data S6**: Trilineage differentiation of AT-MSCs. **Data S7**: Relative gene expression of differentiated AT-MSCs obtained from 3 donors. **Data S8**: Posttransplant monitoring: body weight, postprandial blood sugar, human and mouse insulin. **Data S9**: Immune cell subsets in blood, spleen and bone marrow.

## Data Availability

The dataset supporting the results of this article is included within the article and its additional files.

## Abbreviations

IPCs: Insulin producing cells
hAT-MSCs: Human adipose tissue-derived stem cells
HLA: Human leukocyte antigen
DM: Diabetes mellitus
hESCs: Human embryonic stem cells
hiPSCs: Human-induced pluripotent stem cells
PECs: Pancreatic progenitor endoderm cells
hBM-MSCs: Human bone marrow mesenchymal stem cells
DMEM-LG: Low-glucose Dulbecco’s modified Eagle’s complete medium
FBS: Fetal bovine serum
EDTA: Ethylenediaminetetraacetic acid
DPBS: Dulbecco's phosphate-buffered saline
PRDX6: Peroxiredoxin 6
STZ: Streptozotocin
*INS*: Insulin
*GCG*: Glucagon
*SST*: Somatostatin
*PDX1*: Pancreatic and duodenal homeobox 1
*NGN3*: Neurogenin3
*RFX6*: Regulatory factor X6
*NEUROD1*: Neurogenic differentiation factor 1
*MAFA* & *MAFB*: Musculoaponeurotic fibrosarcoma oncogene homologue A and B
*PAX4*: Paired box 4
*GCK*: Glucokinase
*GLUT-2*: Glucose transporter solute carrier family member 2
*NES*: Nestin
*ESRR*_*γ*_: Estrogen-related receptor gamma
*GAPDH*: Glyceraldehyde-3-phosphate dehydrogenase
IgG: Immunoglobulin G
PE: Phycoerythrin
APC: Allophycocyanin
FITC: Fluorescein isothiocyanate
DAB: Diaminobenzidine
Prkdc^scid^: Protein kinase DNA activated, catalytic subunit
IL-2rg^tm^: Interleukin-2 receptor gamma Chain
NK: Natural killer
HSCs: Hematopoietic stem cells
GM-CSF: Granulocyte macrophage-colony stimulating Factor
IL-3: Interleukin 3

## References

[1] Ghoneim MA, Refaie AF, Elbassiouny BL, Gabr MM, Zakaria MM (2020). From mesenchymal stromal/stem cells to insulin-producing cells: progress and challenges. Stem Cell Rev Rep.

[2] Refaie AF, Elbassiouny BL, Kloc M, Sabek OM, Khater SM, Ismail AM (2021). From mesenchymal stromal/stem cells to insulin-producing cells: immunological considerations. Front Immunol.

[3] Le Blanc K, Tammik C, Rosendahl K, Zetterberg E, Ringdén O (2003). HLA expression and immunologic properties of differentiated and undifferentiated mesenchymal stem cells. Exp Hematol.

[4] Bifari F, Lisi V, Mimiola E, Pasini A, Krampera M (2008). Immune modulation by mesenchymal stem cells. Transfus Med Hemotherapy.

[5] Oh SH, Muzzonigro TM, Bae SH, LaPlante JM, Hatch HM, Petersen BE (2004). Adult bone marrow-derived cells trans-differentiating into insulin-producing cells for the treatment of type I diabetes. Lab Invest.

[6] Tang DQ, Cao LZ, Burkhardt BR, Xia CQ, Litherland SA, Atkinson MA (2004). In vivo and in vitro characterization of insulin-producing cells obtained from murine bone marrow. Diabetes.

[7] Chen LB, Jiang XB, Yang L (2004). Differentiation of rat marrow mesenchymal stem cells into pancreatic islet beta-cells. World J Gastroenterol.

[8] Timper K, Seboek D, Eberhardt M, Linscheid P, Christ-Crain M, Keller U (2006). Human adipose tissue-derived mesenchymal stem cells differentiate into insulin, somatostatin, and glucagon expressing cells. Biochem Biophys Res Commun.

[9] Sun Y, Chen L, Hou XG, Hou WK, Dong JJ, Sun L (2007). Differentiation of bone marrow-derived mesenchymal stem cells from diabetic patients into insulin-producing cells in vitro. Chin Med J (Engl).

[10] Gabr MM, Zakaria MM, Refaie AF, Ismail AM, Abou-El-Mahasen MA, Ashamallah SA (2013). Insulin-producing cells from adult human bone marrow mesenchymal stem cells control streptozotocin-induced diabetes in nude mice. Cell Transplant.

[11] Gabr MM, Zakaria MM, Refaie AF, Khater SM, Ashamallah SA, Rashed SA (2020). PRDX6 promotes the differentiation of human mesenchymal stem (stromal) cells to insulin-producing cells. Biomed Res Int.

[12] Moshtagh PR, Emami SH, Sharifi AM (2013). Differentiation of human adipose-derived mesenchymal stem cell into insulin-producing cells: an in vitro study. J Physiol Biochem.

[13] Czubak P, Bojarska-Junak A, Tabarkiewicz J, Putowski L (2014). A modified method of insulin producing cells' generation from bone marrow-derived mesenchymal stem cells. J Diabetes Res.

[14] Khorsandi L, Nejad-Dehbashi F, Ahangarpour A, Hashemitabar M (2015). Three-dimensional differentiation of bone marrow-derived mesenchymal stem cells into insulin-producing cells. Tissue Cell.

[15] Xin Y, Jiang X, Wang Y, Su X, Sun M, Zhang L (2016). Insulin-producing cells differentiated from human bone marrow mesenchymal stem cells in vitro ameliorate streptozotocin-induced diabetic hyperglycemia. PLoS ONE.

[16] Daryabor G, Shiri EH, Kamali-Sarvestani E (2019). A simple method for the generation of insulin producing cells from bone marrow mesenchymal stem cells. In Vitro Cell Dev Biol Anim.

[17] Wang HS, Shyu JF, Shen WS (2011). Transplantation of insulin-producing cells derived from umbilical cord stromal mesenchymal stem cells to treat NOD mice. Cell Transplant.

[18] Wu H, Wen D, Mahato RI (2013). Third-party mesenchymal stem cells improved human islet transplantation in a humanized diabetic mouse model. Mol Ther.

[19] Yang XF, Chen T, Ren LW, Yang L, Qi H, Li FR (2017). Immunogenicity of insulin-producing cells derived from human umbilical cord mesenchymal stem cells. Exp Ther Med.

[20] Hassanin OM, El-Masry TM, Abu-Zahra FA, El-Adawy S, Abdellah AM (2019). Immune-modulatory changes after transplantation therapy of insulin producing cells derived from Wharton's Jelly human umbilical cord-mesenchymal stem cells in diabetes induced rats. Egypt J Immunol.

[21] Mohammadi N, Mardomi A, Hassannia H, Enderami SE, Ranjbaran H, Rafiei A (2020). Mouse bone marrow-derived mesenchymal stem cells acquire immunogenicity concurrent with differentiation to insulin-producing cells. Immunobiology.

[22] Pfaffl MW (2001). A new mathematical model for relative quantification in real-time RT-PCR. Nucleic Acids Res.

[23] Ito M, Hiramatsu H, Kobayashi K, Suzue K, Kawahata M, Hioki K (2002). NOD/SCID/gamma(c)(null) mouse: an excellent recipient mouse model for engraftment of human cells. Blood.

[24] Ito R, Takahashi T, Katano I, Kawai K, Kamisako T, Ogura T (2013). Establishment of a human allergy model using human IL-3/GM-CSF-transgenic NOG mice. J Immunol.

[25] Hayashi K, Kojima R, Ito M (2006). Strain differences in the diabetogenic activity of streptozotocin in mice. Biol Pharm Bull.

[26] Greiner DL, Brehm MA, Hosur V, Harlan DM, Powers AC, Shultz LD (2011). Humanized mice for the study of type 1 and type 2 diabetes. Ann N Y Acad Sci.

[27] Gabr MM, Zakaria MM, Refaie AF, Abdel-Rahman EA, Reda AM, Ali SS (2017). From human mesenchymal stem cells to insulin-producing cells: comparison between bone marrow- and adipose tissue-derived cells. Biomed Res Int.

[28] Strem BM, Hicok KC, Zhu M, Wulur I, Alfonso Z, Schreiber RE (2005). Multipotential differentiation of adipose tissue-derived stem cells. Keio J Med.

[29] Ribeiro A, Laranjeira P, Mendes S, Velada I, Leite C, Andrade P (2013). Mesenchymal stem cells from umbilical cord matrix, adipose tissue and bone marrow exhibit different capability to suppress peripheral blood B, natural killer and T cells. Stem Cell Res Ther.

[30] Rajagopal J, Anderson WJ, Kume S, Martinez OI, Melton DA (2003). Insulin staining of ES cell progeny from insulin uptake. Science.

[31] Yoshihara E, Wei Z, Lin CS, Fang S, Ahmadian M, Kida Y (2016). ERRγ is required for the metabolic maturation of therapeutically functional glucose-responsive β cells. Cell Metab.

[32] Mori A, Murata S, Tashiro N, Tadokoro T, Okamoto S, Otsuka R (2021). Establishment of human leukocyte antigen-mismatched immune responses after transplantation of human liver bud in humanized mouse models. Cells.

[33] Sun C, Xinzhi L, Liu L, Canet MJ, Guan Y, Fan Y (2016). Effect of fasting time on measuring mouse blood glucose level. Int J Clin Exp Med.

[34] Gabr MM, Zakaria MM, Refaie AF, Khater SM, Ashamallah SA, Ismail AM (2015). Differentiation of human bone marrow-derived mesenchymal stem cells into insulin-producing cells: evidence for further maturation in vivo. Biomed Res Int.

[35] Naeim F, Nagesh Rao P, Song SX, Phan RT, Naeim F, Nagesh RP, Song SX, Phan RT (2018). chapter 2 - principles of immunophenotyping. Atlas of hematopathology.

[36] McBride JA, Striker R (2017). Imbalance in the game of T cells: what can the CD4/CD8 T-cell ratio tell us about HIV and health?. PLoS Pathog.

[37] Yoshihara E, O’Connor C, Gasser E, Wei Z, Oh TG, Tseng TW (2020). Immune-evasive human islet-like organoids ameliorate diabetes. Nature.

