# Supplementary material for: Transplantation of insulin-producing cells derived from human mesenchymal stromal/stem cells into diabetic humanized mice
Source: Stem Cell Res Ther. 2022 Jul 26;13:350. doi: 10.1186/s13287-022-03048-y (PMC9327173; doi:10.1186/s13287-022-03048-y)
Supplement: Supplementary file 1 — Additional file 1. Data S1: Donor HLA typing. Data S2: List of human gene-specific primers used in real time PCR. Data S3: Quantitation of Insulin- and c-peptide-positive cells by flow cytometry. Data S4: Preparation of samples for quantitation of immune cells by flow cytometry. Data S5: Immunophenotyping of donor cells. Data S6: Trilineage differentiation of AT-MSCs. Data S7: Relative gene expression of differentiated AT-MSCs obtained from 3 donors. Data S8: Posttransplant monitoring: body weight, postprandial blood sugar, human and mouse insulin. Data S9: Immune cell subsets in blood, spleen and bone marrow. [file 13287_2022_3048_MOESM1_ESM.docx]

**Supplementary data (1)**

**Donor’s HLA typing**

|  | **HLA-A** | | **HLA-B** | | **HLA-DR** | |
| --- | --- | --- | --- | --- | --- | --- |
| **Donor 1** | 3 | 29 | 27 | 45 | 4 | 11 |
| **Donor 2** | 1 | 26 | 18 | 41 | 7 | 11 |
| **Donor 3** | 1 | 24 | 41 | 44 | 4 | 7 |

**Supplementary data (2)**


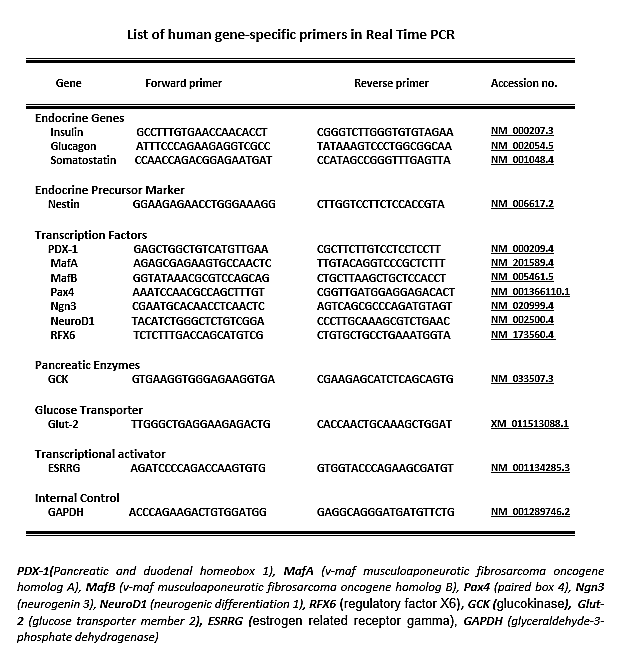


**Supplementary data (3)**

*Quantitation of insulin and c-peptide positive cells by flow cytometry:*

At the end of differentiation 1×10^6^ cells were collected in αMEM (alpha minimum essential media) (Sigma Aldrich St. Louis, MO, USA) containing 10µl of Brefeldin A (Sigma) and incubated at 37˚c in a 5% CO_2_ incubator for 4 hours. The cell preparation was centrifuged at 1200 RPM for 10 minutes, the supernatant was discarded, and the cell pellet was re-suspended in 2ml of Phosphate Buffer Saline (PBS) (Sigma) followed by centrifugation at 1200 RPM for 10 min at 4°C. The cells were than fixed by incubation with 4% paraformaldehyde solution (Thermo Fisher Scientific, Waltham, US) in a concentration of 1 ml/10^6^ cells for 30 min on ice in a dark place. Thereafter, the cells were washed by 2ml PBS once & centrifugated at 1200 RPM at for 10 min at 4°C.  Permeabilization was carried out by intracellular permeabilization solution [Tritone X (Thermo Fisher) + Fetal Bovine Serum (Hyclon, Logan, UT, USA) + PBS (Sigma)] in a concentration of 1ml/10^6^ cells and incubated for 30 min on ice in a dark place. The cells were washed again with intracellular permeabilization solution and centrifuged at 1200 RPM for 10 min at 4°C. The supernatant was discarded, and 10 µL of diluted primary monoclonal antibodies (1:200) for insulin and c-peptide were added to the cell pellet and incubated overnight at 4°c. Cells were washed by 2 ml of intracellular permeabilization solution and centrifuged at 1200 RPM for 10 min at 4°C and the supernatant was discarded. Ten µL of diluted Secondary antibody (1:1000) were added to the cell pellet for 30-60 min on ice in a dark place. The cells were then washed by 500 µL stain buffer (Becton, Dickinson, San Jose, CA, USA) once, centrifuged at 1200 RPM for 10 min at 4°C and the supernatant was discarded. Finally, the cells were re-suspended in 500 µL stain buffer.

**Supplementary data (4)**

***Preparation of samples for quantitation of immune cells by flow cytometry:***

*Whole blood samples (isolation of mononuclear cells):*

One ml of whole blood was collected via cardiac puncture in a heparinized 1cm syringe. Red blood cells were lysed by incubation with 10 mL of 1x ammonium-chloride-potassium (ACK) buffer (Thermo Fisher, Waltham, Massachusetts, USA) for 10 minutes at RT. Thereafter, the cells were spun down at 350 xg for 5 minutes, then the supernatant was discarded, and the cell pellet was washed by stain buffer (Becton, Dickinson, San Jose, CA, USA) to remove any RBCs residuals.

*Isolation of splenocytes:*

After euthanization, the spleen was aseptically removed and immediately placed into a petri dish with 5 mL Hank’s balanced salt solution (HBSS) buffer. Then spleen was then minced by applying pressure in a cell strainer and washed with 5 ml phosphate-buffered saline (PBS)-1mM EDTA. Released splenocytes were centrifuged at 500 xg for 5 minutes at 4°C and the supernatant was discarded. The cell pellet was resuspended in 3 ml ice-cold PBS-EDTA buffer and incubated for 5 minutes on ice. Subsequently, the cell suspension was washed twice with 10 mL cold PBS and centrifuged at 500 xg for 5 minutes at 4°C.

*Isolation of bone marrow hematopoietic cells:*

After euthanization, bone marrow (BM) was obtained from the long bones of each mouse. Briefly, the ends of the bones were cut, and bone marrow was extruded by injecting (Dulbecco’s modified Eagle’s medium containing 10% fetal bovine serum (Sigma) through the bone shaft. The effluent of BM cells was collected and cultured in T-25 flasks. The cells were incubated in Co_2_ incubator at 37^o^C for 2 hr. The nonadherent cells were collected and washed with fresh serum-free medium and centrifuged at 350 xg for 5 minutes.

**Supplementary data (5)**

**Immunophenotyping of donor’s cells**

|  | **CD90%** | **CD105%** | **CD73%** | **CD14%** | **CD45%** | **CD34%** |
| --- | --- | --- | --- | --- | --- | --- |
| **Donor 1** | 99.3 | 96.6 | 98.4 | 0.2 | 0.8 | 0.1 |
| **Donor 1** | 97.3 | 96.4 | 98.7 | 0.0 | 0.0 | 0.1 |
| **Donor 2** | 94.4 | 96 | 98.1 | 0.3 | 0.0 | 0.0 |
| **Donor 2** | 92 | 93 | 97.1 | 0.2 | 0.0 | 0.2 |
| **Donor 3** | 96.8 | 96.7 | 98.7 | 0.0 | 0.0 | 0.0 |
| **Donor 3** | 97.8 | 93.2 | 97.9 | 0.2 | 0.0 | 0.0 |

**Supplementary Data (6)**

**Trilineage differentiation of AT-MSCs**

.

Adipogenesis was detected using Oil-Red-O staining (A), chondrogenesis was detected using Alcian blue (B) and osteogenesis was detected using alizarin-red staining (C).

**
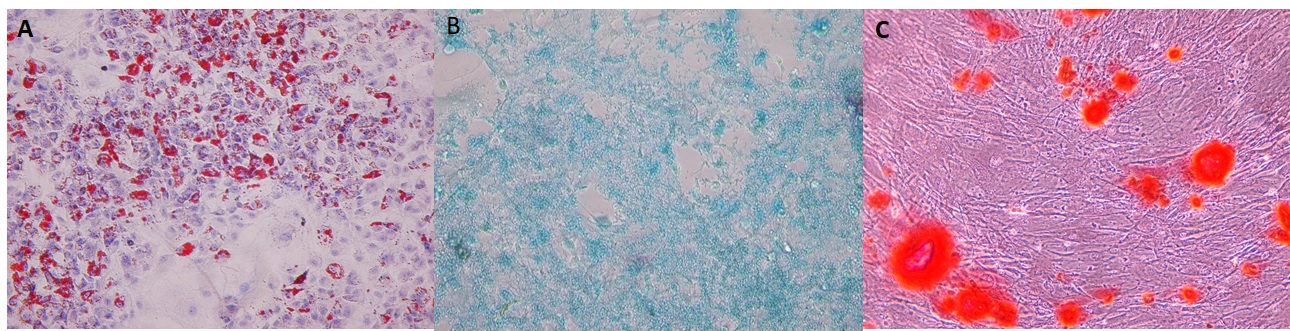
**

**Supplementary Data (7)**

**Relative Gene expression of differentiated AT-MSCs obtained from 3 donors**

|  | **Undifferentiated**  **cells** | **In vitro diff.**  **cells**  **Donor (1)** | **In vitro diff.**  **cells**  **Donor (1)** | **In vitro diff.**  **cells**  **Donor (2)** | **In vitro diff.**  **cells**  **Donor (2)** | **In vitro diff.**  **cells**  **Donor (3)** | **In vitro diff.**  **cells**  **Donor (3)** | **Median values** |
| --- | --- | --- | --- | --- | --- | --- | --- | --- |
| ***INS*** | 1.00 | 6.13 | 6.34 | 3.82 | 5.84 | 4.63 | 4.43 | **4.6** |
| ***GCG*** | 1.00 | 6.52 | 6.61 | 2.60 | 3.73 | 1.39 | 1.34 | **2.6** |
| ***SST*** | 1.00 | 19.23 | 23.18 | 4.86 | 5.98 | 6.02 | 4.69 | **5.9** |
| ***PDX1*** | 1.00 | 4.91 | 5.15 | 3.92 | 5.24 | 3.01 | 2.75 | **3.92** |
| ***NES*** | 1.00 | 4.07 | 4.91 | 1.23 | 1.17 | 2.01 | 2.12 | **2** |
| ***MafA*** | 1.00 | 8.43 | 8.14 | 2.50 | 2.66 | 8.82 | 8.88 | **8.14** |
| ***MafB*** | 1.00 | 4.58 | 4.77 | 4.82 | 5.35 | 6.25 | 5.80 | **4.82** |
| ***GCK*** | 1.00 | 5.70 | 8.40 | 4.06 | 3.89 | 3.31 | 2.78 | **3.8** |
| ***Glut-2*** | 1.00 | 5.46 | 5.54 | 4.16 | 4.52 | 1.49 | 1.55 | **4.16** |
| ***NeruroD1*** | 1.00 | 6.36 | 6.54 | 3.36 | 3.41 | 1.87 | 1.94 | **3.36** |
| ***RFX6*** | 1.00 | 5.82 | 5.62 | 3.29 | 3.76 | 1.44 | 1.46 | **3.29** |
| ***Ngn3*** | 1.00 | 6.30 | 5.68 | 3.49 | 4.21 | 2.07 | 2.06 | **3.49** |
| ***PAX4*** | 1.00 | 1.59 | 1.57 | 6.94 | 7.86 | 1.64 | 1.87 | **1.64** |
| ***ESRRɣ*** | 1.00 | 6.48 | 5.96 | 3.56 | 5.28 | 1.59 | 1.56 | **3.56** |

**Supplementary Data (8)**

**Humanized / Diabetic, Transplanted group**

**Weight, median values (gm)**

| **UNC ID/**  **TACONIC ID** | **Basal** | **Post-induction**  **Of DM**  **Basal** | **Week**  **(1)** | **Week**  **(2)** | **Week**  **(3)** | **Week**  **(4)** | **Week**  **(5)** | **Week**  **(6)** | **Week**  **(7)** | **Week**  **(8)** |
| --- | --- | --- | --- | --- | --- | --- | --- | --- | --- | --- |
| **13/11** | 22 | 18 | 19 | 19 | 18 | 19 | 18 | Died | -- | -- |
| **41/21** | 24 | 21 | 22 | 22 | 22 | 22 | 21 | 21 | 21 | 21 |
| **42/25** | 20 | 22 | 20 | 21 | 21 | 20 | 20 | 20 | 21 | 22 |
| **44/27** | 22 | 20 | 20 | 20 | 19 | 21 | 21 | 21 | 20 | 21 |
| **45/42** | 17 | 25 | 23 | 25 | 25 | 25 | 25 | 25 | 25 | 26 |
| **52/13** | 19 | 20 | 18 | 20 | 20 | 19 | 20 | 19 | 19 | -- |
| **53/5** | -- | 19 | 18 | 19 | 19 | 20 | 19 | 20 | 19 | -- |
| **55/8** | -- | 22 | 22 | 20 | 23 | 22 | 23 | 22 | Died | -- |
| **56/2** | -- | 20 | 19 | 20 | 20 | 20 | 20 | 20 | 20 | -- |
| **Median** | 21.0000 | 20.0000 | 20.0000 | 20.0000 | 20.0000 | 20.0000 | 20.0000 | 20.0000 | 21.5000 | 21.0000 |
| **Min.** | 17.00 | 18.00 | 18.00 | 19.00 | 18.00 | 19.00 | 18.00 | 19.00 | 21.00 | 17.00 |
| **Max.** | 24.00 | 25.00 | 23.00 | 25.00 | 25.00 | 25.00 | 25.00 | 25.00 | 26.00 | 24.00 |

**Humanized / Non-Diabetic, Transplanted mice**

**Weight, median values (gm)**

| **UNC ID/**  **TACONIC ID** | **Basal** | **Week**  **(1)** | **Week**  **(2)** | **Week**  **(3)** | **Week**  **(4)** | **Week**  **(5)** | **Week**  **(6)** | **Week**  **(7)** | **Week**  **(8)** |
| --- | --- | --- | --- | --- | --- | --- | --- | --- | --- |
| **25/21** | 19 | 19 | 21 | 20 | 19 | 20 | 16 | Died | -- |
| **27/26** | 26 | 20 | 20 | 19 | 19 | 21 | 19 | 19 | 17 |
| **28/28** | 20 | 19 | 20 | 21 | 20 | 20 | 19 | 18 | 18 |
| **29/33** | 20 | 20 | 18 | 19 | 18 | 18 | 18 | 19 | 19 |
| **31/46** | 22 | 21 | 22 | 22 | 22 | 22 | 22 | 21 | 21 |
| **Median** | 20.0000 | 20.0000 | 20.0000 | 20.0000 | 19.0000 | 20.0000 | 19.0000 | 19.0000 | 18.5000 |
| **Min.** | 19.00 | 19.00 | 18.00 | 19.00 | 18.00 | 18.00 | 16.00 | 18.00 | 17.00 |
| **Max.** | 26.00 | 21.00 | 22.00 | 22.00 | 22.00 | 22.00 | 22.00 | 21.00 | 21.00 |

**Humanized / Normal Control Groups**

**Weight, median values (gm)**

| **UNC ID/**  **TACONIC ID** | **Basal** | **Week**  **(1)** | **Week**  **(2)** | **Week**  **(3)** | **Week**  **(4)** | **Week**  **(5)** | **Week**  **(6)** | **Week**  **(7)** | **Week**  **(8)** |
| --- | --- | --- | --- | --- | --- | --- | --- | --- | --- |
| **31/46** | 22 | 23 | 23 | 22 | Transplanted | -- | -- | -- | -- |
| **32/43** | 24 | 24 | 25 | 25 | Transplanted  Then died | -- | -- | -- | -- |
| **33/45** | 20 | 21 | 21 | 20 | 21 | 20 | 20 | 19 | 18 |
| **34/26** | 22 | 22 | 22 | 21 | 22 | 22 | 21 | 21 | 22 |
| **46/1** | 17 | 21 | 22 | 22 | 21 | 22 | 22 | 23 | 22 |
| **48/3** | 19 | 19 | 20 | 20 | 19 | 20 | 20 | 20 | 20 |
| **Median** | 21.0000 | 21.5000 | 22.0000 | 21.5000 | 21.0000 | 21.0000 | 20.5000 | 20.5000 | 21.0000 |
| **Min.** | 17.00 | 19.00 | 20.00 | 20.00 | 19.00 | 20.00 | 20.00 | 19.00 | 18.00 |
| **Max.** | 24.00 | 24.00 | 25.00 | 25.00 | 22.00 | 22.00 | 22.00 | 23.00 | 22.00 |

**Diabetic non-Transplanted mice**

**Weight, median values (gm)**

| **UNC ID/**  **TACONIC ID** | **Basal** | **Post-induction**  **Of DM**  **Basal** | **Week**  **(1)** | **Week**  **(2)** | **Week**  **(3)** |
| --- | --- | --- | --- | --- | --- |
| **1** | 21 | 19 | 16 | -- | -- |
| **2** | 22 | 22 | 21 | 18 | -- |
| **3** | 21 | 20 | -- | -- | -- |
| **4** | 20 | 18 | 15 | -- | -- |
| **5** | 23 | 22 | 21 | 18 | -- |
| **Median** | 21 | 20 | 18.5 | 18 | -- |

**Humanized / Diabetic, Transplanted Groups**

**Post prandial blood sugar PPBS, median values (mg/dl)**

| **UNC ID/**  **TACONIC ID** | **Basal** | **Post-induction**  **Of DM**  **Basal** | **Week**  **(1)** | **Week**  **(2)** | **Week**  **(3)** | **Week**  **(4)** | **Week**  **(5)** | **Week**  **(6)** | **Week**  **(7)** | **Week**  **(8)** |
| --- | --- | --- | --- | --- | --- | --- | --- | --- | --- | --- |
| **13/11** | 86.0 | 185 | 110 | 156 | 142 | 114 | 110 | 163 | Died | -- |
| **41/21** | 114.0 | 247 | 130 | 123 | 141 | 122 | 168 | 137 | 168 | 134 |
| **42/25** | 86.0 | 256 | 138 | 113 | 126 | 136 | 127 | 122 | 159 | 121 |
| **44/27** | 104.0 | 218 | 165 | 105 | 125 | 145 | 144 | 150 | 117 | 147 |
| **45/42** | 121.0 | 261 | 163 | 122 | 161 | 182 | 160 | 164 | 165 | 165 |
| **52/13** | 88.0 | 248 | 138 | 173 | 144 | 123 | 128 | 119 | 145 | -- |
| **53/5** | 71.0 | 241 | 147 | 98 | 145 | 112 | 123 | 143 | 143 | -- |
| **55/8** | -- | 238 | 152 | 139 | -- | 108 | 142 | 148 | Died | -- |
| **56/2** | -- | 232 | 142 | 160 | 149 | 134 | 149 | 140 | 130 | -- |
| **Median** | 88.0000 | 241.0000 | 142.0000 | 123.0000 | 142.0000 | 123.0000 | 142.0000 | 143.0000 | 145.0000 | 140.5000 |
| **Min.** | 71.00 | 185.00 | 110.00 | 98.00 | 125.00 | 108.00 | 110.00 | 119.00 | 117.00 | 121.00 |
| **Max.** | 121.00 | 261.00 | 165.00 | 173.00 | 161.00 | 182.00 | 168.00 | 164.00 | 168.00 | 165.00 |

**Humanized / Non-Diabetic, Transplanted mice**

**Post prandial blood sugar, median values (mg/dl)**

| **UNC ID/**  **TACONIC ID** | **Basal** | **Week**  **(1)** | **Week**  **(2)** | **Week**  **(3)** | **Week**  **(4)** | **Week**  **(5)** | **Week**  **(6)** | **Week**  **(7)** | **Week**  **(8)** |
| --- | --- | --- | --- | --- | --- | --- | --- | --- | --- |
| **25/21** | 151 | 155 | 110 | 144 | 123 | 168 | 127 | Died | -- |
| **27/26** | 140 | 126 | 128 | 146 | 149 | 114 | 155 | 131 | 166 |
| **28/28** | 157 | 127 | 125 | 132 | 150 | 152 | 164 | 166 | 176 |
| **29/33** | 122 | 116 | 115 | 145 | 96 | 132 | 132 | 169 | 132 |
| **31/46** | 120 | 146 | 95 | 105 | 112 | 126 | 132 | 124 | 125 |
| **Median** | 140.0000 | 127.0000 | 115.0000 | 144.0000 | 123.0000 | 132.0000 | 132.0000 | 148.5000 | 149.0000 |
| **Min.** | 120.00 | 116.00 | 95.00 | 105.00 | 96.00 | 114.00 | 127.00 | 124.00 | 125.00 |
| **Max.** | 157.00 | 155.00 | 128.00 | 146.00 | 150.00 | 168.00 | 164.00 | 169.00 | 176.00 |

**Humanized / Normal Control Groups**

**Post prandial blood sugar PPBS, median values (mg/dl)**

| **UNC ID/**  **TACONIC ID** | **Basal** | **Week**  **(1)** | **Week**  **(2)** | **Week**  **(3)** | **Week**  **(4)** | **Week**  **(5)** | **Week**  **(6)** | **Week**  **(7)** | **Week**  **(8)** |
| --- | --- | --- | --- | --- | --- | --- | --- | --- | --- |
| **31/46** | 86 | 118 | 112 | 120 | Transplanted | -- | -- | -- | -- |
| **32/43** | 114 | 116 | 126 | 138 | Transplanted  Then died | -- | -- | -- | -- |
| **33/45** | 86 | 111 | 120 | 94 | 133 | 101 | 137 | 119 | 101 |
| **34/26** | 104 | 128 | 101 | 109 | 128 | 115 | 109 | 115 | 140 |
| **46/1** | 121 | 157 | 132 | 128 | 160 | 138 | 125 | 133 | 130 |
| **47/2** | 88 | Died | -- | -- | -- | -- | -- | -- | -- |
| **48/3** | 71 | 130 | 123 | 90 | 112 | 132 | 113 | 124 | 117 |
| **Median** | 88.0000 | 123.0000 | 121.5000 | 114.5000 | 130.5000 | 123.5000 | 119.0000 | 121.5000 | 123.5000 |
| **Min.** | 71.00 | 111.00 | 101.00 | 90.00 | 112.00 | 101.00 | 109.00 | 115.00 | 101.00 |
| **Max.** | 121.00 | 157.00 | 132.00 | 138.00 | 160.00 | 138.00 | 137.00 | 133.00 | 140.00 |

**Diabetic non-Transplanted mice**

**Post prandial blood sugar, median values (mg/dl)**

| **UNC ID/**  **TACONIC ID** | **Basal** | **Post-induction**  **Of DM**  **Basal** | **Week**  **(1)** | **Week**  **(2)** | **Week**  **(3)** |
| --- | --- | --- | --- | --- | --- |
| **1** | 118 | 382 | 420 | -- | -- |
| **2** | 117 | 276 | 384 | 592 | -- |
| **3** | 116 | 405 | -- | -- | -- |
| **4** | 115 | 314 | 585 | -- | -- |
| **5** | 102 | 276 | 385 | 592 | -- |
| **Median** | **116** | **314** | **402** | **592** | **--** |

**Humanized / Diabetic, Transplanted Group**

**Human insulin, median values (µ IU/ml)**

| **UNC ID/**  **TACONIC ID** | **Basal** | **Post-induction**  **Of DM**  **Basal** | **Week**  **(1)** | **Week**  **(2)** | **Week**  **(3)** | **Week**  **(4)** | **Week**  **(5)** | **Week**  **(6)** | **Week**  **(7)** | **Week**  **(8)** |
| --- | --- | --- | --- | --- | --- | --- | --- | --- | --- | --- |
| **13/11** | -- | -- | 7.72 | 4.5 | 3.2 | 3.8 | 3.3 | 3.2 | Died | -- |
| **41/21** | -- | -- | 4.05 | 3.9 | 5.1 | 6.6 | 5.26 | 6.6 | 5.5 | 5.5 |
| **42/25** | -- | -- | 4.2 | 4.0 | 5.38 | 3.9 | 4.7 | 6.5 | 5.3 | 3.1 |
| **44/27** | -- | -- | 4.33 | 4.5 | 5.31 | 4.0 | 5.45 | 6.4 | 5.4 | 3.4 |
| **45/42** | -- | -- | 3.62 | 3.8 | 7.1 | 4.4 | 4.5 | 6.1 | 5.4 | 3.2 |
| **52/13** | -- | -- | 4.6 | 6.8 | 6.9 | 5.3 | 6.5 | 5.0 | 5.2 | -- |
| **53/5** | -- | -- | 5.3 | 6.5 | 6.01 | 5.36 | 6.0 | 5.7 | 5.9 | -- |
| **55/8** | -- | -- | 4.43 | 5.9 | 6.3 | 5.7 | 5.7 | 6.5 | Died | -- |
| **56/2** | -- | -- | 3.82 | 6.0 | 5.9 | 5.0 | 6.2 | 5.55 | 5.8 | -- |
| **Median** |  |  | 4.3300 | 4.5000 | 5.9000 | 5.0000 | 5.4500 | 6.1000 | 5.4000 | 3.3000 |
| **Min.** |  |  | 3.62 | 3.80 | 3.20 | 3.80 | 3.30 | 3.20 | 5.20 | 3.10 |
| **Max.** |  |  | 7.72 | 6.80 | 7.10 | 6.60 | 6.50 | 6.60 | 5.90 | 5.50 |

**Humanized / Non-Diabetic, Transplanted mice**

**Human Insulin, median values (µIU/ml)**

| **UNC ID/**  **TACONIC ID** | **Basal** | **Week**  **(1)** | **Week**  **(2)** | **Week**  **(3)** | **Week**  **(4)** | **Week**  **(5)** | **Week**  **(6)** | **Week**  **(7)** | **Week**  **(8)** |
| --- | --- | --- | --- | --- | --- | --- | --- | --- | --- |
| **25/21** | 0.0 | 1.5 | 2.2 | 1.21 | --- | 0.75 | --- | Died | --- |
| **27/26** | 0.0 | 4.7 | 2.8 | 1.84 | 2.9 | 0.96 | --- | 1.32 | 4.75 |
| **28/28** | 0.0 | 2.75 | --- | 1.4 | 4.9 | 0.83 | 4.4 | 1.45 | 5.03 |
| **29/33** | 0.0 | 1.6 | 2.5 | 1.3 | --- | 0.75 | 4.1 | 3.72 | --- |
| **31/46** | 0.6 | 1.2 | 1.7 | 1.8 | 4.2 | 2.45 | 4.4 | 4.1 | 4.7 |
| **58/36** | 0.0 | 2.5 | 2.25 | 4.6 | --- | 4.38 | 4.1 | 2.9 | 4.43 |
| **61/17** | 0.0 | 1.6 | --- | 2.7 | 4.0 | 4.2 | 4.3 | 3.3 | --- |
| **62/39** | 0.0 | 1.7 | --- | 3.6 | 4.1 | 3.1 | --- | 1.56 | 4.43 |
| **Median** | .0000 | 1.6500 | 2.2500 | 1.8200 | 4.1000 | 1.7050 | 4.3000 | 2.9000 | 4.7000 |
| **Min.** | .00 | 1.20 | 1.70 | 1.21 | 2.90 | .75 | 4.10 | 1.32 | 4.43 |
| **Max.** | .60 | 4.70 | 2.80 | 4.60 | 4.90 | 4.38 | 4.40 | 4.10 | 5.03 |

**Humanized / Normal Control Group**

**Human Insulin, median values (µIU/ml)**

| **UNC ID/**  **TACONIC ID** | **Basal** | **Week**  **(1)** | **Week**  **(2)** | **Week**  **(3)** | **Week**  **(4)** | **Week**  **(5)** | **Week**  **(6)** | **Week**  **(7)** | **Week**  **(8)** |
| --- | --- | --- | --- | --- | --- | --- | --- | --- | --- |
| **33/45** | 0.0 | 0.01 | 0.0 | 0.0 | 0.01 | 0.0 | 0.0 | 0.0 | 0.0 |
| **34/26** | 0.1 | 0.0 | 0.1 | 0.0 | 0.02 | 0.0 | 0.0 | 0.01 | 0.0 |
| **46/1** | 0.02 | 0.0 | 0.0 | 0.0 | 0.0 | 0.0 | 0.0 | 0.0 | 0.0 |
| **48/3** | 0.0 | 0.03 | 0.01 | 0.0 | 0.0 | 0.0 | 0.0 | 0.0 | 0.0 |
| **59/46** | 0.0 | 0.0 | 0.0 | 0.0 | 0.0 | 0.0 | 0.0 | 0.0 | 0.0 |
| **60/43** | 0.01 | 0.0 | 0.0 | 0.0 | 0.0 | 0.0 | 0.0 | 0.0 | 0.0 |
| **Median** | .0050 | .0000 | .0000 | .0000 | .0000 | .0000 | .0000 | .0000 | .0000 |
| **Min.** | .00 | .00 | .00 | .00 | .00 | .00 | .00 | .00 | .00 |
| **Max.** | .10 | .03 | .10 | .00 | .02 | .00 | .00 | .01 | .00 |

**Diabetic non-Transplanted mice**

**Human insulin, median values (µ IU/ml)**

| **UNC ID/**  **TACONIC ID** | **Basal** | **Post-induction**  **Of DM**  **Basal** | **Week**  **(1)** | **Week**  **(2)** | **Week**  **(3)** |
| --- | --- | --- | --- | --- | --- |
| **1** | 0.0 | 0.0 | 0.0 | -- | -- |
| **2** | 0.0 | 0.0 | 0.0 | 0.0 | -- |
| **3** | 0.0 | 0.0 | -- | -- | -- |
| **4** | 0.0 | 0.0 | 0.0 | -- | -- |
| **5** | 0.0 | 0.0 | 0.0 | 0.0 | -- |
| **Median** | **0.0** | **0.0** | **0.0** | **0.0** | **--** |

**Humanized / Diabetic, Transplanted Group**

**Mouse insulin, median values (µ IU/ml)**

| **UNC ID/**  **TACONIC ID** | **Basal** | **Post-induction**  **Of DM**  **Basal** | **Week**  **(1)** | **Week**  **(2)** | **Week**  **(3)** | **Week**  **(4)** | **Week**  **(5)** | **Week**  **(6)** | **Week**  **(7)** | **Week**  **(8)** |
| --- | --- | --- | --- | --- | --- | --- | --- | --- | --- | --- |
| **41/21** | 6.0 | 3.01 | 1.5 | 1.3 | 1.2 | 1.1 | 1.1 | 1.2 | 1.2 | 1.3 |
| **42/25** | 7.6 | 1.53 | 0.9 | 0.8 | 0.6 | 0.6 | 0.5 | 0.4 | 0.4 | 0.4 |
| **44/27** | 6.4 | 2.06 | 1.2 | 1.1 | 1.1 | 1.1 | 1.1 | 0.9 | 0.8 | 0.9 |
| **45/42** | 7.2 | 0.7 | 0.2 | 0.5 | 0.4 | 0.4 | 0.3 | 0.3 | 0.4 | 0.4 |
| **52/13** | 6.4 | 0.6 | 0.8 | 0.7 | 1.2 | 0.2 | 1.1 | 0.4 | 1.1 | 0.4 |
| **53/5** | 7.2 | 1.2 | 0.2 | 1.1 | 0.5 | 0.6 | 0.3 | 0.4 | 0.5 | 0.3 |
| **55/8** | 6.55 | 1.3 | 1.5 | 0.6 | 1.4 | 0.3 | 0.6 | 0.9 | 0.7 | 0.4 |
| **56/2** | 7.6 | 0.5 | 1.2 | 0.5 | 0.8 | 0.6 | 0.6 | 1.2 | 0.3 | 0.9 |
| **Median** | 6.8750 | 1.2500 | 1.0500 | .7500 | .9500 | .6000 | .6000 | .6500 | .6000 | .4000 |
| **Min.** | 6.00 | .50 | .20 | .50 | .40 | .20 | .30 | .30 | .30 | .30 |
| **Max.** | 7.60 | 3.01 | 1.50 | 1.30 | 1.40 | 1.10 | 1.10 | 1.20 | 1.20 | 1.30 |

**Humanized / Non-Diabetic, Transplanted mice**

**mouse Insulin, median values (µIU/ml)**

| **UNC ID/**  **TACONIC ID** | **Basal** | **Week**  **(1)** | **Week**  **(2)** | **Week**  **(3)** | **Week**  **(4)** | **Week**  **(5)** | **Week**  **(6)** | **Week**  **(7)** | **Week**  **(8)** |
| --- | --- | --- | --- | --- | --- | --- | --- | --- | --- |
| **25/21** | 9.6 | --- | 5.9 | --- | 7.2 | --- | 7.4 | Died | --- |
| **27/26** | 8.1 | 2.7 | 1.6 | 3.6 | 6.0 | 3.4 | 6.8 | --- | 3.16 |
| **28/28** | 8.0 | -- | 1.7 | 3.7 | 5.13 | 3.5 | 6.1 | 3.3 | 0.73 |
| **29/33** | 8.0 | 5.05 | 1.6 | --- | 5.0 | 3.8 | 5.15 | 3.48 | 2.1 |
| **31/46** | 7.6 | 3.75 | 3.66 | 4.2 | 3.22 |  | 0.9 | 3.3 | 1.22 |
| **58/36** | 6.55 | 3.52 | 2.94 | 4.0 | 3.4 | 3.5 | 3.6 |  | 3.2 |
| **61/17** | 6.4 | 3.61 | 3.43 | 4.1 | 3.3 |  | 3.6 | 3.57 | 3.35 |
| **62/39** | 7.2 | -- | 3.24 | -- | 5.1 | 3.7 | 3.1 | 4.5 | 3.1 |
| **Median** | 7.8000 | 3.1350 | 3.0900 | 3.7000 | 5.0500 | 3.5000 | 4.3750 | 3.3900 | 3.1000 |
| **Min.** | 6.40 | 1.50 | 1.60 | 1.21 | 3.22 | .75 | .90 | 1.32 | .73 |
| **Max.** | 9.60 | 5.05 | 5.90 | 4.20 | 7.20 | 3.80 | 7.40 | 4.50 | 3.35 |

**Humanized / Normal Control Group**

**Mouse Insulin, median values (µIU/ml)**

| **UNC ID/**  **TACONIC ID** | **Basal** | **Week**  **(1)** | **Week**  **(2)** | **Week**  **(3)** | **Week**  **(4)** | **Week**  **(5)** | **Week**  **(6)** | **Week**  **(7)** | **Week**  **(8)** |
| --- | --- | --- | --- | --- | --- | --- | --- | --- | --- |
| **33/45** | 7.5 | 8.3 | 8.5 | 9.4 | 6.8 | 5.6 | 8.0 | 6.0 | 5.5 |
| **34/26** | 9.1 | 7.1 | 8.8 | 7.0 | 6.9 | 7.1 | 8.2 | 5.8 | 6.0 |
| **46/1** | 7.61 | 6.2 | 8.0 | 4.9 | 6.9 | 8.0 | 7.0 | 8.0 | 7.8 |
| **48/3** | 7.66 | 6.0 | 7.36 | 5.3 | 6.4 | 6.6 | 6.91 | 7.3 | 7.0 |
| **59/46** | 6.3 | 6.0 | 5.67 | 7.0 | 7.026 | 7.95 | 6.0 | 7.29 | 6.24 |
| **60/43** | 6.0 | 6.5 | 6.0 | 6.8 | 6.58 | 7.4 | 7.2 | 7.17 | 6.51 |
| **Median** | 7.5550 | 6.3500 | 7.6800 | 6.9000 | 6.8500 | 7.2500 | 7.1000 | 7.2300 | 6.3750 |
| **Min.** | 6.00 | 6.00 | 5.67 | 4.90 | 6.40 | 5.60 | 6.00 | 5.80 | 5.50 |
| **Max.** | 9.10 | 8.30 | 8.80 | 9.40 | 7.03 | 8.00 | 8.20 | 8.00 | 7.80 |

**Diabetic non-Transplanted mice**

**Mouse insulin, median values (µ IU/ml)**

| **UNC ID/**  **TACONIC ID** | **Basal** | **Post-induction**  **Of DM**  **Basal** | **Week**  **(1)** | **Week**  **(2)** | **Week**  **(3)** |
| --- | --- | --- | --- | --- | --- |
| **1** | 6.9 | 2.0 | 1.8 | -- | -- |
| **2** | 6.0 | 3.0 | 1.7 | 1.4 | -- |
| **3** | 7.4 | 1.4 | -- | -- | -- |
| **4** | 6.8 | 2.1 | 1.2 | -- | -- |
| **5** | 6.0 | 3.0 | 1.4 | 1.2 | -- |
| **Median** | **6.8** | **2.0** | **1.5** | **1.3** | **--** |

**Supplementary Data (9)**

**Immune Cell-subsets from Bone Marrow NOG-EXL-Mice**

| **Normal Control** | | | | | | | | | | |
| --- | --- | --- | --- | --- | --- | --- | --- | --- | --- | --- |
| **UNC/Taconic** | **CD45^+^** | ***CD45^+^,CD19^+^*** | ***CD45^+^,CD19^-^,CD3^+^*** | ***CD45^+^,CD19-,CD3^+^,CD4^+^*** | ***CD45^+^,CD19^-^,***  ***CD3^+^,CD8^+^*** | ***CD45^+^,CD19^-^,***  ***CD3^-^,CD16^+^*** | ***CD45^+^,CD69^+^*** | ***CD45^+^, HLA-ABC^+^*** | ***CD45^+^,HLA-DR^+^*** |  |
| **33/45** | 52.9 | 79.0 | 27.8 | --- | 12.9 | 18.4 | 5.7 | 90.6 | 83.9 |  |
| **34/26** | 44.5 | 66.5 | 29.8 | 75.1 | 11.9 | 13.8 | 8.6 | 88.1 | 82.2 |  |
| **46/1** | 82.2 | 57.5 | 6.0 | 83.5 | 7.5 | 3.2 | 5.7 | 98.6 | 68.8 |  |
| **48/3** | 73.0 | 45.8 | 5.9 | 78.4 | 10.5 | 2.1 | 6.5 | 98.9 | 65.0 |  |
| **60/43** | 47.4 | 43.4 | 22.8 | 65.7 | 29.4 | 0.9 | 7.8 | 96.1 | 67.8 |  |
| **Median** | **52.9** | **57.5** | **22.8** | **76.75** | **11.9** | **3.2** | **6.5** | **96.1** | **68.8** |  |
| **Min.** | **44.50** | **43.40** | **5.90** | **65.70** | **7.50** | **.90** | **5.70** | **88.10** | **65.00** |  |
| **Max.** | **82.20** | **79.00** | **29.80** | **83.50** | **29.40** | **18.40** | **8.60** | **98.90** | **83.90** |  |
| **Non Diabetic transplanted** | | | | | | | | | | |
| **UNC/Taconic** | **CD45+** | ***CD45^+^, CD19^+^*** | ***CD45^+^, CD19^-^, CD3^+^*** | ***CD45^+^, CD19^-^, CD3^+^, CD4^+^*** | ***CD45^+^,CD19^-^, CD3^+^, CD8^+^*** | ***CD45^+^, CD19^-^, CD3^+^, CD16^+^*** | ***CD45^+^, CD69^+^*** | ***CD45^+^, HLA-ABC^+^*** | ***CD45^+^, HLA-DR^+^*** |  |
| **27/26** | 36.2 | 32.4 | 22.7 | 55.3 | 13.6 | 15.7 | 3.8 | 90.7 | 83.5 |  |
| **28/28** | 71.7 | 65.5 | 7.7 | 77.4 | 16.5 | 10.7 | 6.3 | 97 | 94.4 |  |
| **29/33** | 75.2 | 77.4 | 6.9 | 75 | 14.4 | 16.2 | 0.6 | 96.7 | 97 |  |
| **31/46** | 60.4 | 30.5 | 7.3 | 83 | 12.4 | 9.2 | 5.9 | 94.4 | 64.8 |  |
| **58/36** | 57.4 | 39.7 | 16.9 | 84.1 | 14.4 | 2.7 | 4.4 | 98.3 | 88.4 |  |
| **61/17** | 50.4 | ---- | 17.8 | 72 | 23.5 | 3.8 | 4.4 | 88.1 | 75.8 |  |
| **62/39** | 65 | 36.4 | 12.4 | 75.3 | 18.8 | 2.9 | 3.4 | 97.9 | 91.8 |  |
| **Median** | **60.4000** | **38.0500** | **12.4000** | **75.3000** | **14.4000** | **9.2000** | **4.4000** | **96.7000** | **88.4000** |  |
| **Min.** | **36.20** | **30.50** | **6.90** | **55.30** | **12.40** | **2.70** | **.60** | **88.10** | **64.80** |  |
| **Max.** | **75.20** | **77.40** | **22.70** | **84.10** | **23.50** | **16.20** | **6.30** | **98.30** | **97.00** |  |
| **Diabetic transplanted** | | | | | | | | | | |
| **UNC/Taconic** | **CD45+** | ***CD45+,CD19+*** | ***CD45+,CD19***  ***,CD3+*** | ***CD45+,CD19-,CD3+,CD4+*** | ***CD45+,CD19-***  ***,CD3+,CD8+*** | ***CD45+,CD19-,***  ***CD3-,CD16+*** | ***CD45+,CD69+*** | ***CD45+, HLA-ABC+*** | ***CD45+,HLA-DR+*** |  |
| **13/11** | 62.1 | ---- | 15.2 | 69.6 | --- | 14.8 | 2.1 | 88.2 | 77.9 |  |
| **41/21** | 62.5 | 51.5 | 22.7 | 73.3 | 17 | 3.8 | 11.4 | 82.6 | 74.1 |  |
| **42/25** | 63.3 | 34.1 | 21.3 | 86.5 | 8.2 | 2.9 | 12.9 | 72 | 71.6 |  |
| **44/27** | 88.5 | 49.7 | 10.1 | 67.8 | 15.2 | 8.8 | 6.8 | 95.7 | 79.1 |  |
| **45/42** | 89.3 | 30.8 | 10.5 | 86.4 | 6.3 | 5.7 | 10.2 | 94.7 | 76.1 |  |
| **52/13** | 69 | 25.9 | 11.2 | 85.5 | 10.5 | 5.9 | 7.2 | 98.2 | 66.5 |  |
| **53/5** | 80.9 | 39.4 | 5.9 | 81.6 | 12.2 | 6.7 | 4.2 | 97.9 | 74 |  |
| **56/2** | 73.2 | 44.2 | 5.7 | 90.1 | 6.9 | 5.5 | 4 | 96.1 | 79.2 |  |
| **Median** | **71.1000** | **39.4000** | **10.8500** | **83.5500** | **10.5000** | **5.8000** | **7.0000** | **95.2000** | **75.1000** |  |
| **Min.** | **62.10** | **25.90** | **5.70** | **67.80** | **6.30** | **2.90** | **2.10** | **72.00** | **66.50** |  |
| **Max.** | **89.30** | **51.50** | **22.70** | **90.10** | **17.00** | **14.80** | **12.90** | **98.20** | **79.20** |  |
| **P-values** | **0.13** | **0.11** | **0.59** | **0.46** | **0.09** | **0.75** | **0.09** | **0.59** | **0.12** |  |

**Supplementary Data (9)**

**Immune Cell-subsets from blood NOG-EXL-Mice**

| **Normal Control** | | | | | | | | | |
| --- | --- | --- | --- | --- | --- | --- | --- | --- | --- |
| **UNC/Taconic** | **CD45^+^** | ***CD45^+^,CD19^+^*** | ***CD45^+^,CD19^-^,CD3^+^*** | ***CD45^+^,CD19-,CD3^+^,CD4^+^*** | ***CD45^+^,CD19^-^,***  ***CD3^+^,CD8^+^*** | ***CD45^+^,CD19^-^,***  ***CD3^-^,CD16^+^*** | ***CD45^+^,CD69^+^*** | ***CD45^+^, HLA-ABC^+^*** | ***CD45^+^,HLA-DR^+^*** |
| **34/26** | 45.6 | 33.6 | 72.6 | 82.8 | 12.4 | 8.3 | 4.5 | 89.8 | 48.0 |
| **46/1** | 61.6 | 59.2 | 73.3 | 76.0 | 18.8 | 2.1 | 2.8 | 98.6 | 68.8 |
| **48/3** | 44.2 | 78.8 | 57.2 | 62.0 | 31.7 | 1.2 | 3.0 | 97.9 | 81.9 |
| **60/43** | 45.8 | 15.7 | 65.5 | 67.0 | 30.1 | 10.9 | 3.1 | 78.8 | 46.2 |
| **Median** | **45.7000** | **46.4000** | **69.0500** | **71.5000** | **24.4500** | **5.2000** | **3.0500** | **93.8500** | **58.4000** |
| **Min.** | **44.20** | **15.70** | **57.20** | **62.00** | **12.40** | **1.20** | **2.80** | **78.80** | **46.20** |
| **Max.** | **61.60** | **78.80** | **73.30** | **82.80** | **31.70** | **10.90** | **4.50** | **98.60** | **81.90** |
| **Non Diabetic transplanted** | | | | | | | | | |
| **UNC/Taconic** | **CD45+** | ***CD45^+^, CD19^+^*** | ***CD45^+^, CD19^-^, CD3^+^*** | ***CD45^+^, CD19^-^, CD3^+^, CD4^+^*** | ***CD45^+^,CD19^-^, CD3^+^, CD8^+^*** | ***CD45^+^, CD19^-^, CD3^+^, CD16^+^*** | ***CD45^+^, CD69^+^*** | ***CD45^+^, HLA-ABC^+^*** | ***CD45^+^, HLA-DR^+^*** |
| **27/26** | 74.8 | --- | --- | 39.9 | 10.3 | 27.1 | 3.8 | 47.7 | 28.6 |
| **28/28** | 23 | 10.8 | 48.8 | 69.2 | 27.2 | 9.2 | 6.5 | --- | --- |
| **29/33** | 30.9 | 87.6 | 18.5 | 62.2 | 31.1 | 3.5 | 0.2 | 97.7 | 92.9 |
| **31/46** | 33.8 | 58.9 | 86.1 | 67.3 | 29.4 | 4.2 | 0.1 | 95.8 | 71.5 |
| **58/36** | 34.9 | 20.1 | 90.3 | 72.1 | 26.5 | 4.5 | 0.2 | 98.6 | 55.1 |
| **61/17** | 56.5 | 45.6 | 90.3 | 78 | 19.1 | 2.9 | 0.4 | 99.5 | 60.8 |
| **Median** | **34.3500** | **45.6000** | **86.1000** | **68.2500** | **26.8500** | **4.3500** | **.3000** | **97.7000** | **60.8000** |
| **Min.** | **23.00** | **10.80** | **18.50** | **39.90** | **10.30** | **2.90** | **.10** | **47.70** | **28.60** |
| **Max.** | **74.80** | **87.60** | **90.30** | **78.00** | **31.10** | **27.10** | **6.50** | **99.50** | **92.90** |
| **Diabetic transplanted** | | | | | | | | | |
| **UNC/Taconic** | **CD45+** | ***CD45^+^, CD19^+^*** | ***CD45^+^, CD19^-^, CD3^+^*** | ***CD45^+^, CD19^-^, CD3^+^, CD4^+^*** | ***CD45^+^,CD19^-^, CD3^+^, CD8^+^*** | ***CD45^+^, CD19^-^, CD3^+^, CD16^+^*** | ***CD45^+^, CD69^+^*** | ***CD45^+^, HLA-ABC^+^*** | ***CD45^+^, HLA-DR^+^*** |
| **41/11** | **58** | **35.6** | **76.2** | **57.3** | **34.5** | **6.6** | **0.7** | **98.8** | **42.9** |
| **42/25** | **80.5** | **48** | **83.6** | **70.8** | **25.4** | **4.3** | **0.6** | **99.1** | **53.9** |
| **44/27** | **50.9** | **14.3** | **36.5** | **64.8** | **22.6** | **25.7** | **3.4** | **89** | **43.1** |
| **45/42** | **63.5** | **17.9** | **42** | **70** | **18.7** | **11.5** | **2.3** | **78.2** | **37.5** |
| **52/13** | **37.6** | **25.9** | **37.3** | **77.9** | **17.1** | **1.4** | **6** | **95** | **47.4** |
| **53/5** | **57** | **49.2** | **61.7** | **66** | **30.6** | **4.3** | **2.8** | **95.7** | **69** |
| **56/2** | **35.7** | **46.5** | **44.7** | **88.2** | **10.2** | **8.5** | **4.7** | **90.4** | **58** |
| **Median** | **57.0000** | **35.6000** | **44.7000** | **70.0000** | **22.6000** | **6.6000** | **2.8000** | **95.0000** | **47.4000** |
| **Min.** | **35.70** | **14.30** | **36.50** | **57.30** | **10.20** | **1.40** | **.60** | **78.20** | **37.50** |
| **Max.** | **80.50** | **49.20** | **83.60** | **88.20** | **34.50** | **25.70** | **6.00** | **99.10** | **69.00** |
| **P-values** | **0.20** | **0.75** | **0.39** | **0.87** | **0.88** | **0.68** | **0.35** | **0.79** | **0.35** |

**Supplementary Data (9)**

**Immune Cell-subsets from Spleen NOG-EXL-Mice**

| **Normal Control** | | | | | | | | | |
| --- | --- | --- | --- | --- | --- | --- | --- | --- | --- |
| **UNC/Taconic** | **CD45+** | ***CD45^+^, CD19^+^*** | ***CD45^+^, CD19^-^, CD3^+^*** | ***CD45^+^, CD19^-^, CD3^+^, CD4^+^*** | ***CD45^+^, CD19^-^, CD3^+^, CD8^+^*** | ***CD45^+^, CD19^-^, CD3^+^, CD16^+^*** | ***CD45^+^, CD69^+^*** | ***CD45^+^, HLA-ABC^+^*** | ***CD45^+^, HLA-DR^+^*** |
| **33/45** | 73.1 | 66.5 | 78.2 | 53.6 | 37.4 | 2.1 | 5.7 | 95.7 | 67.5 |
| **34/26** | 73.0 | 54.1 | 75.3 | 76.4 | 16.8 | 2.7 | 14.5 | 94.6 | 59.7 |
| **46/1** | 86.9 | 57.9 | 48.3 | 62.3 | 26.5 | 0.2 | 4.9 | 99.8 | 67.0 |
| **48/3** | 77.0 | 62.4 | 48.4 | 49.7 | 38.5 | 0.1 | 3.9 | 99.8 | 53.5 |
| **60/43** | 75.0 | 53.4 | 68.6 | 66.3 | 30.5 | 0.3 | 5.9 | 90.4 | 67.2 |
| **Median** | **75.0000** | **57.9000** | **68.6000** | **62.3000** | **30.5000** | **.3000** | **5.7000** | **95.7000** | **67.0000** |
| **Min.** | **73.00** | **53.40** | **48.30** | **49.70** | **16.80** | **.10** | **3.90** | **90.40** | **53.50** |
| **Max.** | **86.90** | **66.50** | **78.20** | **76.40** | **38.50** | **2.70** | **14.50** | **99.80** | **67.50** |
| **Non Diabetic transplanted** | | | | | | | | | |
| **UNC/Taconic** | **CD45^+^** | ***CD45^+^,CD19^+^*** | ***CD45^+^,CD19^-^,CD3^+^*** | ***CD45^+^,CD19-,CD3^+^,CD4^+^*** | ***CD45^+^,CD19^-^,***  ***CD3^+^,CD8^+^*** | ***CD45^+^,CD19^-^,***  ***CD3^-^,CD16^+^*** | ***CD45^+^,CD69^+^*** | ***CD45^+^, HLA-ABC^+^*** | ***CD45^+^,HLA-DR^+^*** |
| **27/26** | 68 | 41 | 64.1 | 59.3 | 18.5 | 2.7 | 2.7 | 92.3 | 76.4 |
| **28/28** | 72.5 | 80.1 | 25.2 | 62.8 | 33.7 | 1.7 | 1 | 99.5 | 90.3 |
| **29/33** | 59.3 | 76.6 | 48.9 | 67.1 | 28.1 | 0.3 | 1.7 | 93.3 | 85.5 |
| **31/46** | 86.6 | 42.6 | 61.5 | 64.6 | 30.3 | 1.8 | 6.1 | 98.9 | 56.1 |
| **58/36** | 77.8 | 27.8 | 53.6 | 73.8 | 24.1 | 0.3 | 7.4 | 96.6 | 36.7 |
| **61/17** | 68.3 | 62.7 | 46.8 | 69.1 | 23.8 | 0.1 | 1.8 | 96.1 | 76.5 |
| **62/39** | 70.7 | 42.6 | 62.1 | 73.7 | 23.8 | 1.1 | 4.5 | 98.5 | 62.9 |
| **Median** | **70.7000** | **42.6000** | **53.6000** | **67.1000** | **24.1000** | **1.1000** | **2.7000** | **96.6000** | **76.4000** |
| **Min.** | **59.30** | **27.80** | **25.20** | **59.30** | **18.50** | **.10** | **1.00** | **92.30** | **36.70** |
| **Max.** | **86.60** | **80.10** | **64.10** | **73.80** | **33.70** | **2.70** | **7.40** | **99.50** | **90.30** |
| **Diabetic transplanted** | | | | | | | | | |
| **UNC/Taconic** | **CD45^+^** | ***CD45^+^,CD19^+^*** | ***CD45^+^,CD19^-^,CD3^+^*** | ***CD45^+^,CD19-,CD3^+^,CD4^+^*** | ***CD45^+^,CD19^-^,***  ***CD3^+^,CD8^+^*** | ***CD45^+^,CD19^-^,***  ***CD3^-^,CD16^+^*** | ***CD45^+^,CD69^+^*** | ***CD45^+^, HLA-ABC^+^*** | ***CD45^+^,HLA-DR^+^*** |
| **13/11** | 60.1 | 55.2 | 58.1 | 73.1 | 20.6 | 2.2 | 2 | 95.3 | 77.2 |
| **41/21** | 66.6 | 57.6 | 75 | 51.4 | 41.2 | 18 | 5.5 | 87.3 | 49.1 |
| **42/25** | 87.3 | 56.7 | 82.1 | 61.3 | 32.8 | 0.5 | 5.1 | 98.4 | 53.5 |
| **44/27** | 84.6 | 54.7 | 76 | 54.8 | 34.7 | 2.1 | 3.4 | 98.8 | 62.2 |
| **45/42** | 85.5 | 42.8 | 73.5 | 64.5 | 26.8 | 1.3 | 8.3 | 99.1 | 57.7 |
| **52/13** | 74 | 57.8 | 57.4 | 66.2 | 27.9 | 1.2 | 5.4 | 98.1 | 66.7 |
| **53/5** | 86.8 | 69.2 | 52.4 | 61.1 | 31 | 0.4 | 5 | 93.1 | 58.6 |
| **56/2** | 11.6 | 49.5 | 39 | 76.7 | 20.7 | 2 | 9.4 | 98.1 | 76.7 |
| **Median** | **79.3000** | **55.9500** | **65.8000** | **62.9000** | **29.4500** | **1.6500** | **5.2500** | **98.1000** | **60.4000** |
| **Min.** | **11.60** | **42.80** | **39.00** | **51.40** | **20.60** | **.40** | **2.00** | **87.30** | **49.10** |
| **Max.** | **87.30** | **69.20** | **82.10** | **76.70** | **41.20** | **18.00** | **9.40** | **99.10** | **77.20** |
| **P-values** | **0.46** | **0.70** | **0.30** | **0.45** | **0.51** | **0.35** | **0.21** | **0.95** | **0.59** |
